# Urine Metabolites Enable Fast Detection of COVID-19 Using Mass Spectrometry

**DOI:** 10.3390/metabo12111056

**Published:** 2022-11-02

**Authors:** Alexandre Varao Moura, Danilo Cardoso de Oliveira, Alex Ap. R. Silva, Jonas Ribeiro da Rosa, Pedro Henrique Dias Garcia, Pedro Henrique Godoy Sanches, Kyana Y. Garza, Flavio Marcio Macedo Mendes, Mayara Lambert, Junier Marrero Gutierrez, Nicole Marino Granado, Alicia Camacho dos Santos, Iasmim Lopes de Lima, Lisamara Dias de Oliveira Negrini, Marcia Aparecida Antonio, Marcos N. Eberlin, Livia S. Eberlin, Andreia M. Porcari

**Affiliations:** 1 MS^4^Life Laboratory of Mass Spectrometry, Health Sciences Postgraduate Program, São Francisco University, Bragança Paulista 12916-900, SP, Brazil; 2 Department of Chemistry, The University of Texas at Austin, Austin, TX 78712, USA; 3 Department of Material Engineering and Nanotechnology, Mackenzie Presbyterian University, São Paulo 01302-907, SP, Brazil; 4 Municipal Department of Health, Bragança Paulista 12916-900, SP, Brazil; 5 Integrated Unit of Pharmacology and Gastroenterology, UNIFAG, Bragança Paulista 12916-900, SP, Brazil; 6 Department of Surgery, Baylor College of Medicine, Houston, TX 77030, USA

**Keywords:** amino acids, COVID-19, diagnostic, metabolomics, urine

## Abstract

The COVID-19 pandemic boosted the development of diagnostic tests to meet patient needs and provide accurate, sensitive, and fast disease detection. Despite rapid advancements, limitations related to turnaround time, varying performance metrics due to different sampling sites, illness duration, co-infections, and the need for particular reagents still exist. As an alternative diagnostic test, we present urine analysis through flow-injection–tandem mass spectrometry (FIA-MS/MS) as a powerful approach for COVID-19 diagnosis, targeting the detection of amino acids and acylcarnitines. We adapted a method that is widely used for newborn screening tests on dried blood for urine samples in order to detect metabolites related to COVID-19 infection. We analyzed samples from 246 volunteers with diagnostic confirmation via PCR. Urine samples were self-collected, diluted, and analyzed with a run time of 4 min. A Lasso statistical classifier was built using 75/25% data for training/validation sets and achieved high diagnostic performances: 97/90% sensitivity, 95/100% specificity, and 95/97.2% accuracy. Additionally, we predicted on two withheld sets composed of suspected hospitalized/symptomatic COVID-19-PCR negative patients and patients out of the optimal time-frame collection for PCR diagnosis, with promising results. Altogether, we show that the benchmarked FIA-MS/MS method is promising for COVID-19 screening and diagnosis, and is also potentially useful after the peak viral load has passed.

## 1. Introduction

SARS-CoV-2 caused the worst pandemic in the last 100 years. Modern-day laboratory medicine was highly impacted by: the need for the implementation of new technologies; the shortage of the workforce and of supplies, equipment overload, and regulatory changes; this being in addition to the emergence of new mutations [1,2,3]. Considering the incessant demand for fast and accurate diagnosis, the critical role of clinical laboratory tests in human health has become apparent [4,5]. The increasing need for patient testing motivated many clinical laboratories to explore different methods for the collection [6,7,8,9,10], handling [11,12,13], and analysis [14,15,16,17,18,19] of samples, along with different specimen types [20,21].

The most commonly implemented methods for COVID-19 diagnosis rely on molecular-based tests for viral RNA detection [22,23]—which are quantitative antigen tests based on enzyme immunoassays for saliva or nasopharyngeal swabs [24]—or serological tests for the purposes of anti-SARS-CoV-2 immunoglobulin detection [25]. Although the COVID-19 pandemic has accelerated the microbial diagnostics field, accurate and fast diagnosis of SARS-CoV-2 still has several limitations. Some drawbacks are the slow turnaround time, the varying performance of the tests according to sample site, illness duration, the presence of co-infections [26], and the need for particular reagents.

With the expected endemic circulation of the virus, finding new tools for COVID-19 diagnosis remains necessary as virus surveillance may require tests in routine situations such as going to work, school, or large gatherings. The constant threat of new pathogens is an additional motivation for new diagnostic strategies [27]. Moreover, while most tests rely on analyzing nasopharyngeal swabs (NPS), using other biological samples, particularly non-invasive samples, is appealing to facilitate sample collection. Although NPS is a relatively well-tolerated technique, the discomfort level of its collection may vary according to the patient’s age and sex [28,29]. Training and anatomical knowledge are also necessary for NPS collection, as it strongly impacts test sensitivity [30]. Moreover, the implementation of more comfortable and less invasive samples may lead to a higher adherence of individuals to routine testing [31,32]. 

Urine samples can be self-collected and are acquired noninvasively, representing an attractive alternative for non-stressful disease monitoring. Previous studies have explored using urine analysis for COVID-19 diagnosis. For example, in a study by Li et al. using LC-MS—i.e., 25 lipids representing important molecular signatures—were comparatively evaluated across urine and plasma samples, along the course of infection, for the prediction of severity in 30 patients with COVID-19, resulting in a prediction power of 0.904 and 0.988, based on the area under the curve (AUC), for urine and plasma, respectively [20,33]. In a separate study by Bi et al., also using LC-MS, the urinary metabolome was used to confirm altered cytokines and their receptors that are correlated with SARS-CoV-2 replication [34]. Altered urinary metabolomes were also found in COVID-19-infected patients suffering from acute kidney injury (AKI) when compared to healthy controls in 46 subjects. Dewulf et al. compared urine from patients hospitalized with COVID-19 with different degrees of severity against healthy controls using LC-MS and found a significant increase in the levels of tryptophan metabolites [35]. Based on these promising studies, we explored the detection of urinary amino acids and acylcarnitines by flow-injection analysis–tandem mass spectrometry (FIA-MS/MS) as a method for COVID-19 diagnosis [21,36,37,38,39,40,41,42,43,44,45,46,47,48,49,50]. FIA-MS/MS is a method that is widely used to analyze dried blood spots (DBS) and to detect innate errors of metabolism in newborn screenings [51]. The metabolites targeted in this study, amino acids and acylcarnitines, and their relation with COVID-19 infection have also been investigated with MS in different matrices such as plasma [21,36,37,38,39,40,41,42], serum [39,40,43,44,45,47,48,49], and feces [50]. Although FIA-MS/MS is one of the most popular and successful clinical applications of MS, it has not been used for COVID-19 testing, as the totality of the mentioned studies employed chromatographic separation prior to MS detection. 

Here, we demonstrate that urinary amino acids and acylcarnitines are helpful in diagnosing COVID-19 patients based on a cohort of 246 subjects using FIA-MS/MS. We also show that statistical classifiers generated from the metabolic information allow for the diagnosis of COVID-19 with an agreement with PCR of 95%, indicating the utility of this widespread method to be considered as a new screening tool for COVID-19. 

## 2. Materials and Methods

### 2.1. Chemicals

Unlabeled amino acid standards and labeled isovaleryl-DL-carnitine-(N,N,N-trimethyl-d9) hydrochloride were purchased from Merck (Merck KGaA, Darmstadt, Germany). Acetonitrile and methanol HPLC–MS grade solvents were from J.T. Baker.

### 2.2. Subjects

Self-collected urine samples from 246 volunteers were prospectively obtained from July to October 2020 at three medical centers in Bragança Paulista (SP, Brazil); Santa Casa and Bragantino Hospitals; and at the Integrated Unit of Pharmacology and Gastroenterology (UNIFAG). No fasting guidelines were given to the volunteers prior to sample collection. In a convenience sampling, we also recruited healthy volunteers and patients hospitalized with moderate or severe [52] symptoms after being admitted to the medical center. Patients older than 18 years old with suspicion of COVID-19 were recruited according to the following eligibility criteria: patients hospitalized in the medical center, non-pregnant, without mechanical ventilation or indwelling catheter; further, patients who were facing imminent death were excluded. Healthy non-pregnant volunteers older than 18 were selected if they declared no previous contamination by COVID-19 or close contact with infected people. 

Institutional Review Board (IRB) approval was received for the study (protocol number 31573020.9.0000.5514, approved on 29 May 2020). Samples were collected from healthy volunteers (n = 104) and hospitalized volunteers when they possessed symptoms similar to those found in COVID-19 infections (n = 142). All the healthy and symptomatic volunteers had their diagnoses confirmed via an analysis of nasopharyngeal swab samples through an RT-PCR, which were used for the purposes of recruitment into the study or as part of their clinical care, using Brazilian-certified analysis services. RT-PCR was performed using a TaqPath COVID-19 RT-PCR IVD Kit (Thermo Fisher), and the results were interpreted using the COVID-19 Interpretative Software, according to the manufacturer’s instructions, with a cycle threshold (Ct) value of <37. Positive SARS-CoV-2 infection was confirmed for 99 hospitalized volunteers and discarded for 43. Table 1 provides the patient demographic and clinical information. Patients or volunteers with inconclusive RT-PCR results were resampled or excluded.

### 2.3. Sample Preparation

Urine samples were heat-inactivated after collection (65 °C, 30 min) [53] in a Class II biological safety cabinet before being aliquoted and frozen until extraction. All the samples were thawed at room temperature. A pooled sample was prepared from equal parts (10 μL) of each sample and then aliquoted in different quality control (QC) samples, which were extracted and distributed every ten injections for instrumental monitoring. This resulted in 10 QC samples for system suitability and 28 samples QC for intra-batch monitoring. Samples (300 μL) were randomized and centrifuged (12,000 rpm, 4 °C, 10 min). Next, the supernatant (150 μL) was collected, following the addition of water (120 μL), acetonitrile (15 μL), and internal standard (IS) solution (15 μL of isovaleryl-DL-carnitine-(N,N,N-trimethyl-d9) hydrochloride solution at 11.1 ng mL^−1^ in methanol). Blank samples were prepared using ultrapure water instead of urine.

### 2.4. Flow Injection–Tandem MS Analysis

Data acquisition was performed on a Waters^®^ Xevo TQD triple quadrupole mass spectrometer equipped with a Shimadzu^®^ SCL-10A controller, a Shimadzu^®^ LC-20AD pump controller, and a Shimadzu^®^ SIL-20A automatic sampler injector. The methodology employed Flow Injection Analysis (FIA) without chromatographic separation, and 10 µL was used as injection volume. Further, the mobile phase was composed of water:acetonitrile:formic acid (80:20:0.1 *v/v/v*). A flow gradient was used, starting with a zeroed flow until 0.5 min. We initially zeroed the flow rate to allow the integration of the entire peak, with no cuts due to the proximity to the y-axis. Afterward, the flow ranged from 0 to 0.5 mL min^−1^ from 0.5 to 0.51 min, at which point it was maintained until 3.50 min, and was then decreased to 0.1 mL min^−1^, with a total runtime of 4 min. Multiple reaction monitoring (MRM) transitions were optimized for each compound by analyzing labeled and unlabeled standards, as described in Supplementary Table S1 (ST1). The acquisition was controlled by the Target Lynx software (Waters).

### 2.5. Data Analysis and Statistical Classifiers

The ratio of the peak areas of the analytes and the IS was considered and processed using Metaboanalyst 5.0 (http://www.metaboanalyst.ca) [54]. Calculations were made based on the relative peak area ratios of each analyte/IS through the different groups. Missing values were replaced by 1/5 of the minimal positive values of their corresponding variables. Relative standard deviation (RSD) was calculated for the intra-batch QC samples, and those analytes found with RSD > 25% were not considered for statistical modeling. Interquartile range filtering was applied in order to remove variables with near-constant values. Data normalization was performed by sum, followed by generalized logarithm transformation [55], while the Pareto scaling method was applied. The resulting dataset was used for statistical analysis using the least absolute shrinkage and selection operator (Lasso). 

As hospitalized patients had their urine samples collected in a time lapse from 0 to 95 days from the swab collection to RT-PCR diagnosis, we, therefore, used a time frame to select patients in order to build the statistical classifier. For this purpose, we considered time-qualified samples, such as those from volunteers with a time interval of two days between urine and swab collections and the onset of symptoms of 14 days or less from the urine collection. The classifier was built using 75% of data from healthy non-hospitalized COVID-19 PCR-negative (n = 78, Neg-NH) and hospitalized COVID-19 PCR-positive (n = 32, Pos-H) patients. We validated the model with the remaining 25% of the data composed of Pos-H (n = 10) and Neg-NH (n= 26) volunteers. Additionally, we tested the ability of this model to predict on a withheld sample set (Withheld Set 1) composed of suspected hospitalized/symptomatic COVID-19 PCR-negative (n = 24, Neg-H) patients. We also tested this classifier’s prediction on samples that were excluded because they did not meet the selected time interval criteria for swab collection/symptoms onset. This sample set (Withheld Set 2) was composed of Pos-H (n = 57) and suspected hospitalized/symptomatic Neg-H (n = 19) patients. Cutoff values for positivity definition were selected based on the receiver operator characteristics (ROC) curve for training and validation sets. We evaluated the model’s performance for the validation and test sets by measuring the predictive accuracy, sensitivity, specificity, negative predictive value (NPV), and positive predictive value (PPV), which were all calculated based on the agreement with PCR diagnosis. 

Univariate analysis was performed after data normalization using the Kruskal–Wallis test for the three groups (Pos-H, Neg-NH, and Neg-H), followed by Dunn’s post hoc test, using the Benjamini–Hochberg (BH) correction for the *p*-value. Afterward, the Mann–Whitney test was used to examine differences between Pos-H vs. Neg-NH, Pos-H vs. Neg-H, and Neg-NH vs. Neg-H (25), followed by the BH correction of the *p*-value. The stability of the analytes to the heat-inactivation process was evaluated using RSD (Supplementary Table S2). Calculations were performed in R version 3.6.3 (R Foundation for Statistical Computing). Discriminant metabolic markers found by Lasso analysis were interrogated for the purposes of pathway enrichment analysis by using the metabolite set enrichment analysis (MSEA) via over-representation analysis from the Metaboanalyst web platform [56]. Two metabolomics databases were interrogated, i.e., Kegg and the MSEA’s disease-associated metabolite sets using urine as a reference (Supplementary Figure S1) [56,57]. 

## 3. Results

Detection of 19 amino acids, such as alanine, leucine, glutamine, tryptophan, and 15 acylcarnitines—such as free-carnitine, malonyl-carnitine, octadecanoyl-carnitine—were achieved from urine analysis, as presented in the Supplementary Table S1 along with the relative standard deviation (RSD) measured for the QC samples (Supplementary Table S2). Although asparagine and aspartate were detected in our method, they were excluded from statistical analyses due to the higher variability measured in their peak area ratios (RSD > 25%, n = 28, ST2). Monitoring the labeled internal standard signal along the QC samples resulted in 3.3% of RSD (N = 28 QC samples, Supplementary Table S2), showcasing the analytical stability of the method. Note that the heat inactivation process did not appear to alter the peak area of the analytes, as the RSD measured between heat-inactivated and non-inactivated samples was lower than 15% for the entire set of analytes (Supplementary Table S2). Twenty-nine metabolites were detected with metrics above thresholds established for RSD and thermal stability (Supplementary Table S3); further, these were then used for statistical analysis.

**Figure 1:**
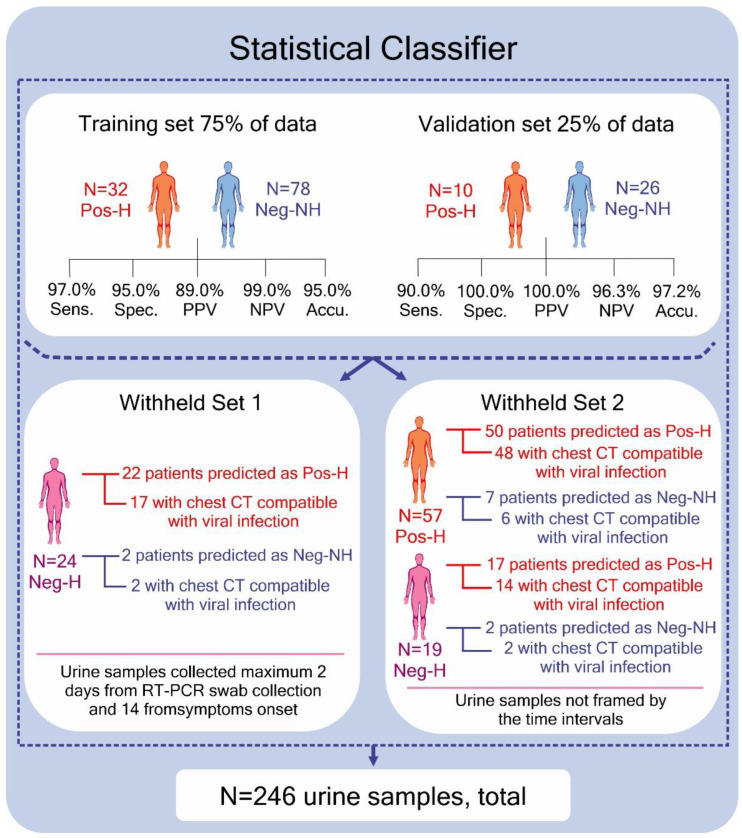
The statistical classifier’s experimental design and performance when identifying healthy non-hospitalized COVID-19 PCR-negative (Neg-NH) volunteers or hospitalized COVID-19 PCR-positive (Pos-H) patients. The Withheld Set 1 was composed of suspected hospitalized/symptomatic COVID-19 PCR-negative (Neg-H) patients. In contrast, the Withheld Set 2 was composed of Pos-H and Neg-H patients who did not meet the time frame criteria.

Figure 1 shows that high diagnostic performances were achieved using statistical analysis for the training and validation sample sets. Only 1 out of 32 Pos-H samples in the training set and 1 out of 10 Pos-H samples from the validation set were erroneously classified as negative, resulting in high sensitivity (97% and 90%) and negative predictive values (NPV) of 99.0% and 96.3% for the training and validation sets, respectively. Amongst negative samples, 4 out of 78 were misclassified in the training set. In contrast, none of the 26 samples were misclassified as positive in the validation set, resulting in positive predictive values (PPV) of 89.0% and 100.0% for the test and validation sets, respectively; further, specificities of 95% and 100% were noted for these sets. The overall agreement to PCR was 95.0% and 97.2% for the test and validation sets, respectively. The cutoff value for classification was 0.181. The influence of age on the classifier’s predictive performance was evaluated and was noted to have minimally improved the classification metrics (Supplementary Table S4). However, we opted not to take this variable into account with the goal of building a model that is independent of age; this is because we expect to adapt the model to different populations in the future. We observed that other studies also reported age and sex disparities in their sample sets, which is one of the disadvantages of using convenience sampling approaches. Dewulf et al. investigated a targeted urinary metabolic panel in 56 patients who were hospitalized with COVID-19 (26 non-critical and 30 critical); further, they also utilized 16 healthy controls and 3 controls with proximal tubule dysfunction unrelated to SARS-CoV-2 [35]. Their control set comprised 31% men, while their positive set comprised 69–83% men. Thomas et al. also reported a divergence in age and sex when evaluating serum metabolites of patients with COVID-19 (n = 33, which was diagnosed by nucleic acid testing), compared with COVID-19–negative controls (n = 16). They reported 76% of subjects in the disease group as male, aged 56.5 ± 18.1 years old (mean ± standard deviation), and a control group comprising 38% of men, aged 37.8 ± 11.6 years old [46]. Ling Yan et al. used the serum peptidome as the diagnostic matrix for COVID-19 [58]. The group infected by COVID-19 had an average age of 46.6 ± 14.9 and 47.2 ± 15.4 (training and validation sets), whereas the control group had an average age of 32.4 ± 11.4 and 29.6 ± 10.2, for training and validation sets. 

When the statistical classifier was used to predict the Withheld Set 1 (Supplementary Table S6A)—which was composed of time-framed hospitalized/symptomatic COVID-19 PCR-negative patients (Neg-H)—22 of the 24 Neg-H samples were classified as positive. Interestingly, when inspecting the clinical data from these patients, 17 (77%) of them presented chest computed tomography (CT) scans with suggestive signals of viral infection, such as ground-glass opacity (GGO), consolidation, and pulmonary commitment [59], despite the negative PCR result. Our model’s two remaining patients, classified as negative, presented a viral infection suggestive chest CT scan.

When inspecting the results for Withheld Set 2, the time lapse between urine sample collection and the RT-PCR test, or days from symptom onset, did not strongly impact the model’s performance as 50 (87.7%) out of 57 Pos-H samples were correctly classified as being positive. Neg-H samples were not considered when calculating, which means the classifier’s performance since the diagnosis for the patients were not conclusive based on their clinical attributes. Nonetheless, 14 of 19 Neg-H samples in Withheld Set 2 were classified according to their chest CT scan findings. To view detailed clinical information of the patients from the Withheld Set 2, see Supplementary Table S6B in the Data Supplement.

**Figure 2:**
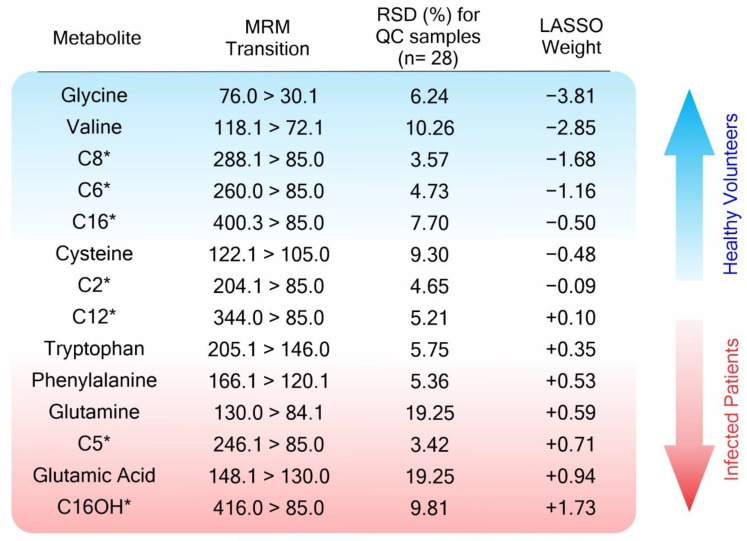
Metabolites selected by Lasso analysis, the multiple reaction monitoring (MRM) transitions of detection (precursor > fragment), relative standard deviation (RSD) for quality control (QC) samples, and their weights for the Lasso model. * Acylcarnitines are expressed by the number of carbons on the chain.

**Figure 3:**
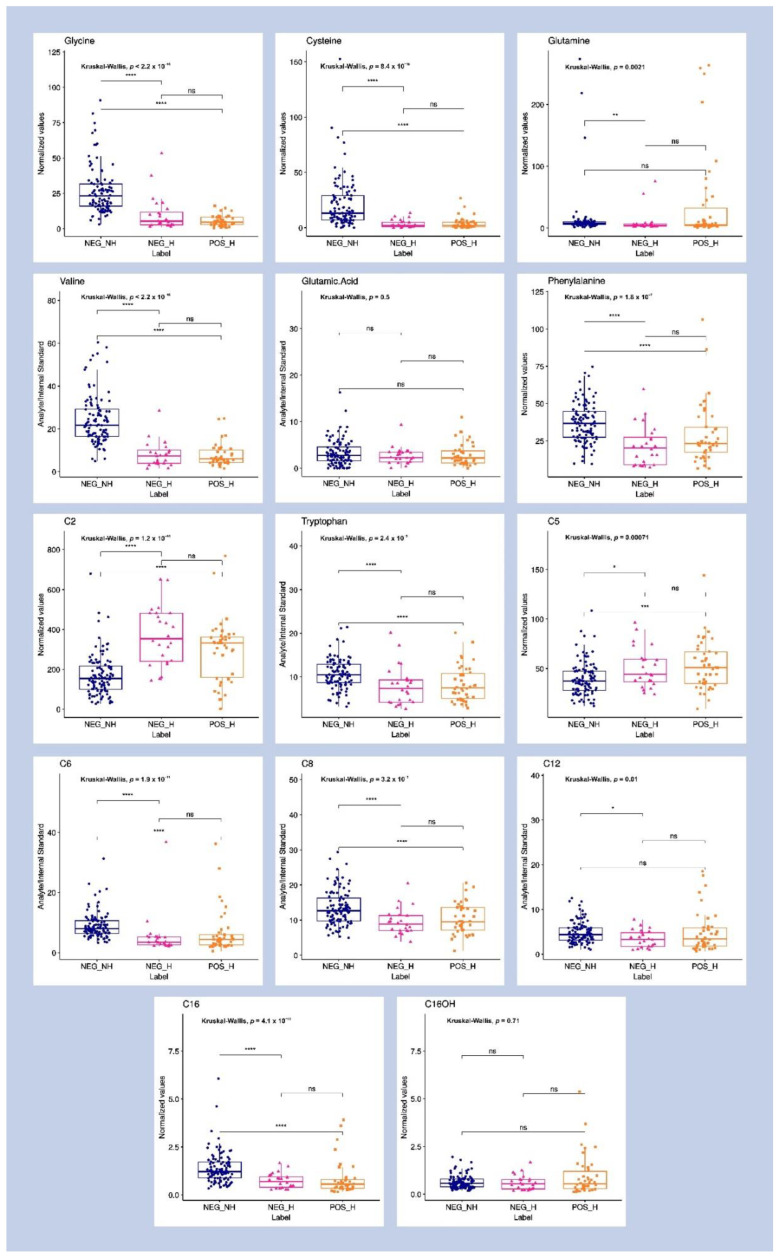
Box plots for the analytes found as discriminatory by the Lasso model and their abundance between the three classes of volunteers: healthy non-hospitalized COVID-19 PCR-negative (Neg-NH) volunteers, hospitalized COVID-19 PCR-positive (Pos-H) patients and suspected hospitalized/symptomatic COVID-19 PCR-negative (Neg-H) patients. Univariate analysis was performed using the Kruskal–Wallis test. If a *p*-value is less than 0.05, it is flagged with one star (*). If a *p*-value is less than 0.01, it is flagged with 2 stars (**). If a *p*-value is less than 0.001, it is flagged with three stars (***). If a *p*-value is less than 0.0001, it is flagged with four stars (****).

The statistical classifier, built using the Lasso algorithms, was based on 14 predictive metabolites, which were given associated mathematical weights according to their relevance to each classifier class, as described in Figure 2. Some variables, which were Lasso selected, also have significant values for the purposes of univariate statistical analysis, such as fold change and adjusted *p*-value, as presented in Supplementary Table S5A. To visualize the changes in metabolite abundance—which are also in the Neg-H group—not accounted for when using the binary Lasso model, we additionally performed a univariate analysis based on the Kruskal–Wallis test (Figure 3 and Supplementary Table S5B). We could not find any significant metabolic alteration when comparing Neg-H and Pos-H groups, which is in agreement with their similar clinical states. On the other hand, 13 of 14 metabolites indicated by the Lasso analysis were also altered between Neg-NH and Neg-H, evidencing how the metabolites are affected by hospitalization and clinical symptoms.

**Figure 4:**
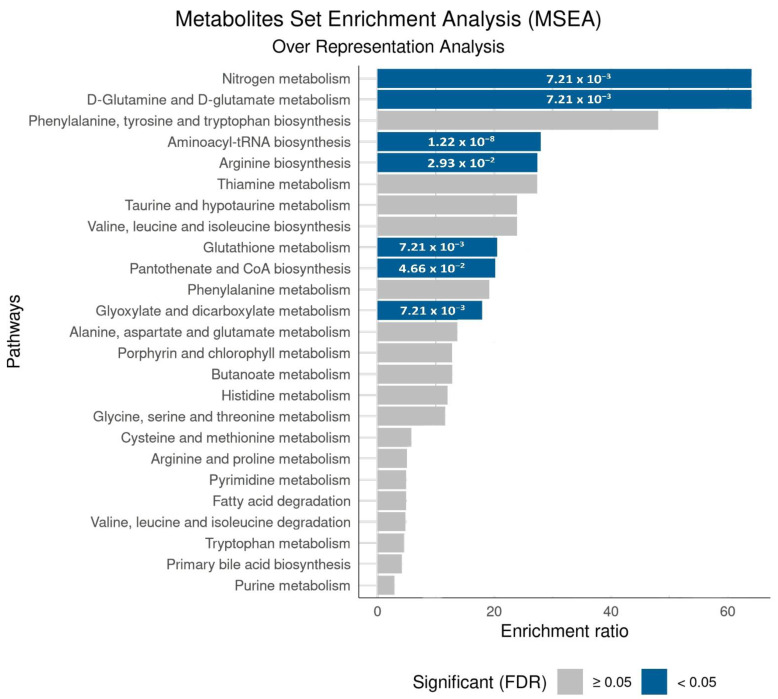
Metabolite enrichment analysis via over representation analysis for the discriminatory analytes found by Lasso analysis. A set of human metabolites from the KEGG library was used, and the *p*-adjusted values for the pathways are presented by the color bar, with the significant ones (FDR < 0.05) displayed numerically.

To investigate the biological significance of the metabolites selected by our model and evaluate if the changes observed in the chemical patterns were correlated to biological processes involved in infections, we performed a metabolite enrichment analysis of the discriminatory analytes. This analysis resulted in seven significantly altered pathways (FDR < 0.05), as shown in Figure 4.

## 4. Discussion

The method we developed for COVID-19 diagnosis is an adaptation of the well-known and worldwide established newborn screening methodology based on selective MS/MS detection. Utilizing a cohort of 246 RT-PCR validated samples, we opted to build a classifier using samples selected based on rigorous criteria that ensured maximum viral load based on the proximity of the onset of symptoms and RT-PCR collection date. Using this approach, we showed that a panel of amino acids and acylcarnitine could be used to develop classification models that are highly sensitive (>90%), specific (>95%), and accurate (>95%) for COVID-19 screening (Figure 1). 

The reported performances of serological or antigen tests for diagnosis or confirmation of SARS-CoV-2 infection present sensitivities ranging from 21.8 to 97.9% (serological) and 34.1 to 96% (antigen), as recently revised by Bastos et al. [25], and Dinnes et al. [60]. These authors revised 104 studies, including 38 serological tests and 16 antigen tests applied to symptomatic volunteers, finding specificities ranging from 80.6 to 100% for serological tests and 34.1 to 96% for antigen tests. Böger et al. reviewed the performance of RT-PCR of nasopharyngeal specimens in four different studies and found 73.3% for sensitivity and 100% for specificity [61]. The method we introduced here presented a simple sample workup consisting of dilution and centrifugation. We developed the method to provide a short processing time, with a run time of 4 min, with no chromatographic separation. Good sensitivities and specificity rates were found, as well as also the ability to detect COVID-19 infection outside the “optimal detection window”, as presented in Figure 1. Altogether, these results showcase the potential of FIA-MS/MS to be used as a screening technique or for time course follow-up. However, further studies for clinical validation should include the evaluation of contamination with other viruses, such as influenza, and including positive asymptomatic people and other virus variants. 

The classification results obtained for the Withheld Set 1, composed of samples from suspect Neg-H patients, suggest that our classifier mainly reflects patient infection status, given the agreement with chest CT scan results (Figure 1). The chest CT scan is a fundamental tool for COVID-19 diagnosing and monitoring. However, it cannot differentiate between an active or previous viral infection or, indeed, indicate the viral pathogen—resulting in lower specificity than RT-PCR for COVID-19 diagnosis [62,63,64,65,66]. For patients from Withheld Set 1, the negative result from the RT-PCR test was in disagreement with their clinical profile and chest CT scan findings for most cases (19 out of 24). From 19 Neg-H patients with viral suggestive chest CT, our model classified 17 as being positive for COVID-19. For example, patient #34 (see Supplementary Table S6A in Data Supplement), a 76-year-old male, received a negative result for RT-PCR, while he was classified as positive by our classifier. The patient presented a chest CT scan that was suggestive of viral infection with ground-glass opacity, consolidations, and pulmonary commitment (50%). The patient was in the intensive care unit (ICU) for 13 days, 11 of which required the use of mechanical ventilation, until death. As recognized by many studies [62,63,64,65], repeated PCR tests should be used for patients with an inconclusive diagnosis in order to more accurately diagnose COVID-19, although repeated PCR tests were not performed for the patients in our study as this could have resulted in a false-negative diagnosis. The disagreement of RT-PCR and chest CT scan results for the Neg-H volunteers, assumed to be the absence of a second-tier or confirmatory test for these individuals, motivated their exclusion from the training/validation sets and also in the option to keep them predicted within Withheld Set 1. The effect of the time lapse between symptoms onset and sample collection day was interrogated by analyzing the Withheld Set 2, which showed similar results to those acquired for training and validation sets. The results suggests that the detected metabolic alterations enabled sample classification for patients who were assessed more than 14 days from symptoms onset and after two days from RT-PCR detection. 

Based on the unveiled altered pathways, the selected molecular panel appears to correlate with systemic molecular changes that are associated with COVID-19 infection (as shown in Figure 4). The altered amino acids were related to alterations in pathways enrolled in processes such as cellular bioenergetics [67,68,69], immune regulation [70,71,72], metabolic changes [73,74,75], oxidative stress [76,77,78], and protein regulation [79,80,81]. Many metabolic alterations were also found to be correlated with amino acid alterations, specifically during COVID-19 infection [67,68,69,70,71,72,73,74,75,76,77,78,79,80,81]. To better correlate our findings to known alterations in the related pathways, we organized Table 2. This table summarizes the altered amino acids, the MSEA-impacted pathway, other related biological processes, and how these pathways and processes might be impacted during COVID-19 infection, according to the literature. For example, glycine urinary levels were decreased in infected volunteers (*p* < 2.2 × 10^−16^). According to MSEA, this alteration significantly impacted pathways such as aminoacyl-tRNA biosynthesis; glyoxylate and dicarboxylate metabolism; and glutathione metabolism. Glycine acts on the regulation of pro-inflammatory cytokines that control immune response [82,83], and an increased level of this amino acid may be related to a decrease in oxidative stress and inflammatory processes [83,84], as these processes were reported during COVID-19 infection [70,71,72,76,77,78]. Another altered amino acid, valine, is a precursor of the cofactor CoA and acts on mitochondria protein transporters [85], which will ultimately impact the release of acylcarnitines.

The Lasso analysis also ranked acylcarnitines as important markers for COVID-19 infection (Figure 2). These molecules act mainly inside the mitochondria during the beta-oxidation process and act as an active acyl-group buffer [96,97], which plays an essential role in cellular bioenergetics [98]. Outside the mitochondrial membrane, the acyl-CoA is formed and enzymatically converted to acyl-carnitine, which passes through the outer mitochondrial membrane; next, it then passes through the inner mitochondrial membrane by the Carnitine:Acylcarnitine Carrier (CAC) antiport protein [96,99,100]. Changes in plasmatic acylcarnitine profiles are directly related to cardiovascular and metabolic syndrome [94,101,102,103], the main comorbidities associated with our sampling set (Table 1, section Comorbidity), and with COVID-19 itself [104,105,106], reflecting the worst outcomes [107]. Long-chain acylcarnitines (C12-C20) accumulate at the air–fluid interface of the lungs in response to stress, such as influenza infection [108]. The fine control and clearance of these metabolites are assigned to the kidneys [96], which could explain their detection in urine.

## 5. Conclusions

In conclusion, we showed that urine analysis, using an adaptation of the known method for newborn screening by FIA-MS/MS, is a promising methodology for COVID-19 screening and diagnosis, with the potential to be used even after the peak viral load passes. The non-invasive sample collection, the lack of need for specific primers, and the possibility of using existing laboratory resources in order to implement the methodology demonstrate the technique’s feasibility to be fully validated. This includes multi-center trials, as well as occurrences for newborn screening programs. Our method also revealed substantial changes in the metabolome of infected patients and pointed out the relation of COVID-19 to other diseases, providing insights into the physiopathology of the disease. Importantly, our method uses urine, a non-invasive and self-collectible sample that would ease the collection procedure without overburdening medical staff. Urine has also been shown to contain dense and consistent biological information regarding COVID-19 infection.

Further advancements should focus on measuring the specificity of the method for samples that are obtained from patients presenting multiple pathogens, as well as its ability to detect COVID-19 in asymptomatic infected people or to distinguish COVID-19 infection from other critical diseases. Longitudinal experiments following the time course of the infection would also be valuable to better understand the metabolic changes in urine during different phases of the infection. The challenges faced in developing new alternatives for COVID-19 screening underscore the need to provide new methodological insights ahead of the next health security crisis.

## Figures and Tables

**Table 1:** Clinic and demographic information of individuals recruited for the study, including SARS-CoV-2-negative non-hospitalized subjects (Neg-NH) and SARS-CoV-2-positive hospitalized subjects (Pos-H), used for model building and evaluation. Withheld Sets 1 and 2 containing symptomatic SARS-CoV-2-negative hospitalized subjects (Neg-H) are also shown.

	Classifier ^a^ (Training + Validation)		Withheld Set 1	Withheld Set 2	
	Neg-NH	Pos-H	Neg-H	Pos-H	Neg-H
Total = 246	104 n (%)	42 n (%)	24 n (%)	57 n (%)	19 n (%)
Age—mean (min–max) ^b^	38.2 (20–89)	56.2 (21–86)	58.8 (26–81)	56.3 (26–77)	59.8 (30–83)
Female ^c^	61 (58.7)	13 (31.0)	11 (45.8)	23 (40.4)	10 (52.6)
Male ^c^	43 (41.3)	29 (69.0)	13 (54.2)	34 (59.6)	9 (47.4)
Symptoms					
Fever	0 (0.0)	23 (54.8)	11 (45.8)	36 (63.2)	10 (52.6)
Cough	0 (0.0)	30 (71.4)	14 (58.3)	35 (61.4)	11 (57.9)
Myalgia	0 (0.0)	8 (19.0)	2 (8.3)	13 (22.8)	3 (15.8)
Sore throat	1 (1.0)	7 (16.7)	8 (33.3)	8 (14.0)	2 (10.5)
Headache	3 (2.9)	12 (28.6)	2 (8.3)	7 (12.3)	5 (26.3)
Coryza	0 (0.0)	5 (11.9)	3 (12.5)	6 (10.5)	2 (10.5)
Dyspnea	0 (0.0)	29 (69.0)	16 (66.7)	29 (50.9)	12 (63.2)
Oxygen saturation < 95%	0 (0.0)	17 (40.5)	10 (41.7)	19 (33.3)	3 (15.8)
Tiredness/fatigue	0 (0.0)	3 (7.1)	2 (8.3)	7 (12.3)	3 (15.8)
Loss of smell or taste	0 (0.0)	8 (19.0)	9 (37.5)	12 (21.1)	5 (26.3)
Vomiting or nausea	0 (0.0)	2 (4.8)	3 (12.5)	9 (15.8)	1 (5.3)
Diarrhea	0 (0.0)	11 (26.2)	2 (8.3)	12 (21.1)	3 (15.8)
Comorbidity					
SAH ^d^	16 (15.4)	21 (50.0)	10 (41.7)	29 (50.9)	8 (42.1)
Cardiovascular disease	2 (1.9)	7 (16.7)	4 (16.7)	10 (17.5)	4 (21.1)
Obesity	12 (11.5)	9 (21.4)	1 (4.2)	13 (22.8)	4 (21.1)
Diabetes mellitus	3 (2.9)	17 (40.5)	2 (8.3)	18 (31.6)	4 (21.1)
Neoplasia	0 (0.0)	3 (7.1)	0 (0.0)	1 (1.8)	1 (5.3)
Lung disease	8 (7.7)	3 (7.1)	6 (25.0)	5 (8.8)	4 (21.1)
COPD ^e^	1 (1.0)	1 (2.4)	2 (8.3)	3 (5.3)	2 (10.5)
Smoker or ex-smoker	6 (5.8)	3 (7.1)	4 (16.7)	3 (5.3)	3 (15.8)
Asthma	2 (1.9)	2 (4.8)	2 (8.3)	2 (3.5)	1 (5.3)
Kidney disease	0 (0.0)	2 (4.8)	0 (0.0)	1 (1.8)	1 (5.3)
Tomography Findings					
Ground glass opacity	0 (0.0)	40 (95.2)	19 (79.2)	54 (94.7)	16 (84.2)
Consolidations	0 (0.0)	20 (47.6)	13 (54.2)	32 (56.1)	8 (42.1)
Crazy-paving appearance	0 (0.0)	19 (45.2)	10 (41.7)	22 (38.6)	8 (42.1)
reticular pattern	0 (0.0)	6 (14.3)	6 (25.0)	16 (28.1)	2 (10.5)
Pulmonary commitment degree	0 (0.0)	35 (83.3)	16 (66.7)	49 (86.0)	12 (63.2)
Suggestive of viral infection	0 (0.0)	40 (95.2)	19 (79.2)	54 (94.7)	16 (84.2)

^a^: Estimated statistical power of 99.2% (alfa = 0.05, Cohen, 1988); ^b^: *p*-value for the age is 2.7 × 10^−6^ for the classifier, and 0.31 for the Withheld Set 2 (Mann–Whitney–Wilcoxon test); ^c^: *p*-value for the sex is 4.4 × 10^−3^ and 0.35 (Xi-square test) for these groups, respectively; ^d^: SAH: systemic arterial hypertension; and ^e^: COPD: chronic obstructive pulmonary disease.

**Table 2:** Amino acids and pathways significantly altered according to the metabolic set enrichment analysis (MSEA), as well as other related biological processes and their reported biological function when considering COVID-19 infection.

Amino Acids	Impacted Pathway (MSEA)	Related Pathway	Impact in COVID-19
Glycine (Gly)	Aminoacyl-tRNA biosynthesis Glyoxylate and dicarboxylate metabolism Glutathione metabolism	Immune regulation [82,83] Oxidative stress [83,84]	[70,71,72,76,77,78]
Valine (Val)	Aminoacyl-tRNA biosynthesis Pantothenate and CoA biosynthesis	Immune regulation [86]	[70,71,72]
Cysteine (Cys)	Aminoacyl-tRNA biosynthesis Pantothenate and CoA biosynthesis	Oxidative stress [82,85,87]; Protein regulation [85,88]	[76,77,78,79,80,81]
Tryptophan (Try)	Aminoacyl-tRNA biosynthesis	Immune regulation [35,41,46,74,89,90]	[70,71,72]
Phenylalanine (Phe)	Aminoacyl-tRNA biosynthesis	Bioenergetics [41,46]; Immune regulation [35,74,89,90]	[67,68,69,70,71,72]
Glutamine (Gln)	Aminoacyl-tRNA biosynthesis D-Glutamine and D-glutamate metabolism Nitrogen metabolism Glyoxylate and dicarboxylate metabolism Arginine biosynthesis	Immune regulation [74,91,92]; Metabolic changes [46,93]; Oxidative stress [94,95]	[70,71,72,73,74,75,76,77,78]
Glutamate (Glu) (glutamic acid)	Aminoacyl-tRNA biosynthesis D-Glutamine and D-glutamate metabolism Nitrogen metabolism Glyoxylate and dicarboxylate metabolism Arginine biosynthesis	Metabolic changes [39,49,93]; Oxidative stress [39,46]	[73,74,75,76,77,78]

## Data Availability

The data analyzed and generated in our work are available upon request from the corresponding author. The data are not publicly available due to patient confidentiality, participant privacy, and ethical restrictions.

## Supplementary Materials

The following supporting information can be downloaded at: https://www.mdpi.com/article/10.3390/metabo12111056/s1. Table S1: Amino acids and acylcarnitines investigated in urine from COVID-19 patients and their experimental detection parameters. Table S2: Relative standard deviation of metabolites and internal standard for QC samples during batch analysis and after the heat inactivation test. Table S3: Amino acids and acylcarntines selected after RSD and IQR filtering processes performed in MetaboAnalyst. Table S4: The effect of age and sex on the model’s performance. Table S5A: Comparison of analytes between the groups (Pos-H and Neg-NH) of patients using the Mann–Whitney test. Table S5B: Comparison analytes between the groups (Pos-H, Neg-NH, and Neg-H) of patients using the Kruskal–Wallis test and Dunn’s Test as a post hoc measure. Table S6A: Classification of the Withheld Set 1 patients and their clinical characteristics. S6B: Classification of the Withheld Set 2 patients and their clinical characteristics. Supporting Figure S1: Metabolite enrichment analysis for the discriminatory analytes found by Lasso analysis; Supporting Figure S2: Metabolite enrichment analysis by over representation analysis for the discriminatory analytes found by Lasso analysis.

## References

[1] Pritt B.S., Wang P., Nuzzo J., Zimmermann S., Burnham C.-A.D. (2022). Deadly Pathogens, Transformative Technologies, and Protracted Pandemics: Challenges and Opportunities in Laboratory Medicine. Clin. Chem..

[2] Fernandes Q., Inchakalody V.P., Merhi M., Mestiri S., Taib N., Moustafa Abo El-Ella D., Bedhiafi T., Raza A., Al-Zaidan L., Mohsen M.O. (2022). Emerging COVID-19 variants and their impact on SARS-CoV-2 diagnosis, therapeutics and vaccines. Ann. Med..

[3] Lima N.M., Fernandes B.L.M., Alves G.F., de Souza J.C.Q., Siqueira M.M., Patrícia do Nascimento M., Moreira O.B.O., Sussulini A., de Oliveira M.A.L. (2022). Mass spectrometry applied to diagnosis, prognosis, and therapeutic targets identification for the novel coronavirus SARS-CoV-2: A review. Anal. Chim. Acta.

[4] Poon L.L.M. (2022). A Push for Real Normal: Mass Screening for COVID-19. Clin. Chem..

[5] Lai C.-C., Shih T.-P., Ko W.-C., Tang H.-J., Hsueh P.-R. (2020). Severe acute respiratory syndrome coronavirus 2 (SARS-CoV-2) and coronavirus disease-2019 (COVID-19): The epidemic and the challenges. Int. J. Antimicrob. Agents.

[6] Utama R., Hapsari R., Puspitasari I., Sari D., Hendrianingtyas M., Nurainy N. (2022). Self-collected gargle specimen as a patient-friendly sample collection method for COVID-19 diagnosis in a population context. Sci. Rep..

[7] Pasomsub E., Watcharananan S.P., Boonyawat K., Janchompoo P., Wongtabtim G., Suksuwan W., Sungkanuparph S., Phuphuakrat A. (2021). Saliva sample as a non-invasive specimen for the diagnosis of coronavirus disease 2019: A cross-sectional study. Clin. Microbiol. Infect..

[8] Liao W.T., Hsu M.Y., Shen C.F., Hung K.F., Cheng C.M. (2020). Home Sample Self-Collection for COVID-19 Patients. Adv. Biosyst..

[9] Petruzzi G., De Virgilio A., Pichi B., Mazzola F., Zocchi J., Mercante G., Spriano G., Pellini R. (2020). COVID-19: Nasal and oropharyngeal swab. Head Neck.

[10] Bwire G.M., Majigo M.V., Njiro B.J., Mawazo A. (2021). Detection profile of SARS-CoV-2 using RT-PCR in different types of clinical specimens: A systematic review and meta-analysis. J. Med. Virol..

[11] Yelin I., Aharony N., Tamar E.S., Argoetti A., Messer E., Berenbaum D., Shafran E., Kuzli A., Gandali N., Shkedi O. (2020). Evaluation of COVID-19 RT-qPCR Test in Multi sample Pools. Clin. Infect. Dis..

[12] Stokes W., Berenger B.M., Scott B., Szelewicki J., Singh T., Portnoy D., Turnbull L., Pabbaraju K., Shokoples S., Wong A.A. (2022). One Swab Fits All: Performance of a Rapid, Antigen-Based SARS-CoV-2 Test Using a Nasal Swab, Nasopharyngeal Swab for Nasal Collection, and RT–PCR Confirmation from Residual Extraction Buffer. J. Appl. Lab. Med..

[13] Gokulan C.G., Kiran U., Kuncha S.K., Mishra R.K. (2021). Temporal stability and detection sensitivity of the dry swab-based diagnosis of SARS-CoV-2. J. Biosci..

[14] Nascimento E.D., Fonseca W.T., de Oliveira T.R., de Correia C., Faça V.M., de Morais B.P., Silvestrini V.C., Pott-Junior H., Teixeira F.R., Faria R.C. (2022). COVID-19 diagnosis by SARS-CoV-2 Spike protein detection in saliva using an ultrasensitive magneto-assay based on disposable electrochemical sensor. Sens. Actuators B Chem..

[15] Garza K.Y., Silva A.A.R., Rosa J.R., Keating M.F., Povilaitis S.C., Spradlin M., Sanches P.H.G., Moura A.V., Gutierrez J.M., Lin J.Q. (2021). Rapid Screening of COVID-19 Disease Directly from Clinical Nasopharyngeal Swabs using the MasSpec Pen Technology. Anal. Chem..

[16] Dhar B.C. (2022). Diagnostic assay and technology advancement for detecting SARS-CoV-2 infections causing the COVID-19 pandemic. Anal. Bioanal. Chem..

[17] Wandtke T., Wędrowska E., Szczur M., Przybylski G., Libura M., Kopiński P. (2022). Aptamers-Diagnostic and Therapeutic Solution in SARS-CoV-2. Int. J. Mol. Sci..

[18] Moore K.J.M., Cahill J., Aidelberg G., Aronoff R., Bektaş A., Bezdan D., Butler D.J., Chittur S.V., Codyre M., Federici F. (2021). Loop-Mediated Isothermal Amplification Detection of SARS-CoV-2 and Myriad Other Applications. J. Biomol. Tech..

[19] Drobysh M., Ramanaviciene A., Viter R., Chen C.F., Samukaite-Bubniene U., Ratautaite V., Ramanavicius A. (2022). Biosensors for the Determination of SARS-CoV-2 Virus and Diagnosis of COVID-19 Infection. Int. J. Mol. Sci..

[20] Li Y., Hou G., Zhou H., Wang Y., Tun H.M., Zhu A., Zhao J., Xiao F., Lin S., Liu D. (2021). Multi-platform omics analysis reveals molecular signature for COVID-19 pathogenesis, prognosis and drug target discovery. Signal. Transduct. Target. Ther..

[21] Barberis E., Timo S., Amede E., Vanella V.V., Puricelli C., Cappellano G., Raineri D., Cittone M.G., Rizzi E., Pedrinelli A.R. (2020). Large-Scale Plasma Analysis Revealed New Mechanisms and Molecules Associated with the Host Response to SARS-CoV-2. Int. J. Mol. Sci..

[22] Sethuraman N., Jeremiah S.S., Ryo A. (2020). Interpreting Diagnostic Tests for SARS-CoV-2. JAMA.

[23] Wang W., Xu Y., Gao R., Lu R., Han K., Wu G., Tan W. (2020). Detection of SARS-CoV-2 in Different Types of Clinical Specimens. JAMA.

[24] Aoki K., Nagasawa T., Ishii Y., Yagi S., Okuma S., Kashiwagi K., Maeda T., Miyazaki T., Yoshizawa S., Tateda K. (2021). Clinical validation of quantitative SARS-CoV-2 antigen assays to estimate SARS-CoV-2 viral loads in nasopharyngeal swabs. J. Infect. Chemother. Off. J. Jpn. Soc. Chemother..

[25] Lisboa Bastos M., Tavaziva G., Abidi S.K., Campbell J.R., Haraoui L.-P., Johnston J.C., Lan Z., Law S., MacLean E., Trajman A. (2020). Diagnostic accuracy of serological tests for covid-19: Systematic review and meta-analysis. BMJ.

[26] Sutjipto S., Lee P.H., Tay J.Y., Mendis S.M., Abdad M.Y., Marimuthu K., Ng O.T., Cui L., Chan M., Soon M. (2020). The Effect of Sample Site, Illness Duration, and the Presence of Pneumonia on the Detection of SARS-CoV-2 by Real-time Reverse Transcription PCR. Open Forum Infect. Dis..

[27] Patel R. (2022). Advances in Testing for Infectious Diseases—Looking Back and Projecting Forward. Clin. Chem..

[28] Li L., Shim T., Zapanta P.E. (2021). Optimization of COVID-19 testing accuracy with nasal anatomy education. Am. J. Otolaryngol..

[29] Marra P., Colacurcio V., Bisogno A., De Luca P., Calvanese M., Petrosino M., De Bonis E., Troisi D., Cassandro C., Cavaliere M. (2021). Evaluation of Discomfort in Nasopharyngeal Swab Specimen Collection for SARS-CoV-2 Diagnosis. Clin. Ter..

[30] Kim D.H., Kim D., Moon J.W., Chae S.W., Rhyu I.J. (2022). Complications of Nasopharyngeal Swabs and Safe Procedures for COVID-19 Testing Based on Anatomical Knowledge. J. Korean Med. Sci..

[31] Lin L., Song Y., Wang Q., Pu J., Sun F.Y., Zhang Y., Zhou X., Larson H.J., Hou Z. (2021). Public Attitudes and Factors of COVID-19 Testing Hesitancy in the United Kingdom and China: Comparative Infodemiology Study. JMIR Infodemiol..

[32] Ehrenstein B., Schwarz T., Fleck M., Günther F. (2021). Hygiene measures against COVID-19 in routine outpatient care: Acceptance by the patients?. Z. Rheumatol..

[33] Li Y., Yao L., Li J., Chen L., Song Y., Cai Z., Yang C. (2020). Stability issues of RT-PCR testing of SARS-CoV-2 for hospitalized patients clinically diagnosed with COVID-19. J. Med. Virol..

[34] Bi X., Liu W., Ding X., Liang S., Zheng Y., Zhu X., Quan S., Yi X., Xiang N., Du J. (2022). Proteomic and metabolomic profiling of urine uncovers immune responses in patients with COVID-19. Cell Rep..

[35] Dewulf J.P., Martin M., Marie S., Oguz F., Belkhir L., De Greef J., Yombi J.C., Wittebole X., Laterre P.-F., Jadoul M. (2022). Urine metabolomics links dysregulation of the tryptophan-kynurenine pathway to inflammation and severity of COVID-19. Sci. Rep..

[36] Su Y., Chen D., Yuan D., Lausted C., Choi J., Dai C.L., Voillet V., Duvvuri V.R., Scherler K., Troisch P. (2020). Multi-Omics Resolves a Sharp Disease-State Shift between Mild and Moderate COVID-19. Cell.

[37] Blasco H., Bessy C., Plantier L., Lefevre A., Piver E., Bernard L., Marlet J., Stefic K., Benz-de Bretagne I., Cannet P. (2020). The specific metabolome profiling of patients infected by SARS-COV-2 supports the key role of tryptophan-nicotinamide pathway and cytosine metabolism. Sci. Rep..

[38] Song J.-W., Lam S.M., Fan X., Cao W.-J., Wang S.-Y., Tian H., Chua G.H., Zhang C., Meng F.-P., Xu Z. (2020). Omics-Driven Systems Interrogation of Metabolic Dysregulation in COVID-19 Pathogenesis. Cell Metab..

[39] Danlos F.-X., Grajeda-Iglesias C., Durand S., Sauvat A., Roumier M., Cantin D., Colomba E., Rohmer J., Pommeret F., Baciarello G. (2021). Metabolomic analyses of COVID-19 patients unravel stage-dependent and prognostic biomarkers. Cell Death Dis..

[40] Gray N., Lawler N.G., Yang R., Morillon A.-C., Gay M.C.L., Bong S.-H., Holmes E., Nicholson J.K., Whiley L. (2021). A simultaneous exploratory and quantitative amino acid and biogenic amine metabolic profiling platform for rapid disease phenotyping via UPLC-QToF-MS. Talanta.

[41] Kimhofer T., Lodge S., Whiley L., Gray N., Loo R.L., Lawler N.G., Nitschke P., Bong S.-H., Morrison D.L., Begum S. (2020). Integrative Modeling of Quantitative Plasma Lipoprotein, Metabolic, and Amino Acid Data Reveals a Multiorgan Pathological Signature of SARS-CoV-2 Infection. J. Proteome Res..

[42] Wu D., Shu T., Yang X., Song J.-X., Zhang M., Yao C., Liu W., Huang M., Yu Y., Yang Q. (2020). Plasma metabolomic and lipidomic alterations associated with COVID-19. Natl. Sci. Rev..

[43] Schwarz B., Sharma L., Roberts L., Peng X., Bermejo S., Leighton I., Casanovas-Massana A., Minasyan M., Farhadian S., Ko A.I. (2021). Cutting Edge: Severe SARS-CoV-2 Infection in Humans Is Defined by a Shift in the Serum Lipidome, Resulting in Dysregulation of Eicosanoid Immune Mediators. J. Immunol..

[44] Doğan H.O., Şenol O., Bolat S., Yıldız Ş.N., Büyüktuna S.A., Sarıismailoğlu R., Doğan K., Hasbek M., Hekim S.N. (2021). Understanding the pathophysiological changes via untargeted metabolomics in COVID-19 patients. J. Med. Virol..

[45] Mohammed A.F.K., Alghetaa H., Miranda K., Wilson K., P. Singh N., Cai G., Putluri N., Nagarkatti P., Nagarkatti M. (2020). Δ9-Tetrahydrocannabinol Prevents Mortality from Acute Respiratory Distress Syndrome through the Induction of Apoptosis in Immune Cells, Leading to Cytokine Storm Suppression. Int. J. Mol. Sci..

[46] Thomas T., Stefanoni D., Reisz J.A., Nemkov T., Bertolone L., Francis R.O., Hudson K.E., Zimring J.C., Hansen K.C., Hod E.A. (2020). COVID-19 infection alters kynurenine and fatty acid metabolism, correlating with IL-6 levels and renal status. JCI Insight.

[47] Shen B., Yi X., Sun Y., Bi X., Du J., Zhang C., Quan S., Zhang F., Sun R., Qian L. (2020). Proteomic and Metabolomic Characterization of COVID-19 Patient Sera. Cell.

[48] Xiao N., Nie M., Pang H., Wang B., Hu J., Meng X., Li K., Ran X., Long Q., Deng H. (2021). Integrated cytokine and metabolite analysis reveals immunometabolic reprogramming in COVID-19 patients with therapeutic implications. Nat. Commun..

[49] Shi D., Yan R., Lv L., Jiang H., Lu Y., Sheng J., Xie J., Wu W., Xia J., Xu K. (2021). The serum metabolome of COVID-19 patients is distinctive and predictive. Metab. Clin. Exp..

[50] Lv L., Jiang H., Chen Y., Gu S., Xia J., Zhang H., Lu Y., Yan R., Li L. (2021). The faecal metabolome in COVID-19 patients is altered and associated with clinical features and gut microbes. Anal. Chim. Acta.

[51] Seger C., Salzmann L. (2020). After another decade: LC-MS/MS became routine in clinical diagnostics. Clin. Biochem..

[52] Wei P.-F. (2020). Diagnosis and Treatment Protocol for Novel Coronavirus Pneumonia (Trial Version 7). Chin. Med. J..

[53] Kampf G., Voss A., Scheithauer S. (2020). Inactivation of coronaviruses by heat. J. Hosp. Infect..

[54] Chong J., Wishart D.S., Xia J. (2019). Using MetaboAnalyst 4.0 for Comprehensive and Integrative Metabolomics Data Analysis. Curr. Protoc. Bioinform..

[55] Durbin B.P., Hardin J.S., Hawkins D.M., Rocke D.M. (2002). A variance-stabilizing transformation for gene-expression microarray data. Bioinformatics.

[56] Xia J., Wishart D.S. (2010). MSEA: A web-based tool to identify biologically meaningful patterns in quantitative metabolomic data. Nucleic Acids Res..

[57] Kanehisa M., Goto S., Kawashima S., Okuno Y., Hattori M. (2004). The KEGG resource for deciphering the genome. Nucleic Acids Res..

[58] Yan L., Yi J., Huang C., Zhang J., Fu S., Li Z., Lyu Q., Xu Y., Wang K., Yang H. (2021). Rapid Detection of COVID-19 Using MALDI-TOF-Based Serum Peptidome Profiling. Anal. Chem..

[59] Kwee T.C., Kwee R.M. (2020). Chest CT in COVID-19: What the Radiologist Needs to Know. Radiographics.

[60] Dinnes J., Deeks J.J., Berhane S., Taylor M., Adriano A., Davenport C., Dittrich S., Emperador D., Takwoingi Y., Cunningham J. (2021). Rapid, point-of-care antigen and molecular-based tests for diagnosis of SARS-CoV-2 infection. Cochrane Database Syst. Rev..

[61] Böger B., Fachi M.M., Vilhena R.O., Cobre A.F., Tonin F.S., Pontarolo R. (2021). Systematic review with meta-analysis of the accuracy of diagnostic tests for COVID-19. Am. J. Infect. Control.

[62] Yang W., Sirajuddin A., Zhang X., Liu G., Teng Z., Zhao S., Lu M. (2020). The role of imaging in 2019 novel coronavirus pneumonia (COVID-19). Eur. Radiol..

[63] Ufuk F., Savaş R. (2020). Chest CT features of the novel coronavirus disease (COVID-19). Turk. J. Med. Sci..

[64] Xie X., Zhong Z., Zhao W., Zheng C., Wang F., Liu J. (2020). Chest CT for Typical Coronavirus Disease 2019 (COVID-19) Pneumonia: Relationship to Negative RT-PCR Testing. Radiology.

[65] Feng H., Liu Y., Lv M., Zhong J. (2020). A case report of COVID-19 with false negative RT-PCR test: Necessity of chest CT. Jpn J. Radiol..

[66] Hossein H., Ali K.M., Hosseini M., Sarveazad A., Safari S., Yousefifard M. (2020). Value of chest computed tomography scan in diagnosis of COVID-19; a systematic review and meta-analysis. Clin. Transl. Imaging.

[67] Gibellini L., De Biasi S., Paolini A., Borella R., Boraldi F., Mattioli M., Lo Tartaro D., Fidanza L., Caro-Maldonado A., Meschiari M. (2020). Altered bioenergetics and mitochondrial dysfunction of monocytes in patients with COVID-19 pneumonia. EMBO Mol. Med..

[68] Surazakov A., Klassen A., Gizinger O. (2020). The bioenergetics of COVID-19 immunopathology and the therapeutic potential of biophysical radiances. J. Photochem. Photobiol. B Biol..

[69] Gvozdjakova A., Klauco F., Kucharska J., Sumbalova Z. (2020). Is mitochondrial bioenergetics and coenzyme Q10 the target of a virus causing COVID-19?. Bratisl. Lek. Listy.

[70] Trombetta A.C., Farias G.B., Gomes A.M.C., Godinho-Santos A., Rosmaninho P., Conceição C.M., Laia J., Santos D.F., Almeida A.R.M., Mota C. (2021). Severe COVID-19 Recovery Is Associated with Timely Acquisition of a Myeloid Cell Immune-Regulatory Phenotype. Front. Immunol..

[71] Yazdanpanah F., Hamblin M.R., Rezaei N. (2020). The immune system and COVID-19: Friend or foe?. Life Sci..

[72] Paces J., Strizova Z., Smrz D., Cerny J. (2020). COVID-19 and the immune system. Physiol. Res..

[73] Atila A., Alay H., Yaman M.E., Akman T.C., Cadirci E., Bayrak B., Celik S., Atila N.E., Yaganoglu A.M., Kadioglu Y. (2021). The serum amino acid profile in COVID-19. Amino. Acids.

[74] Ansone L., Briviba M., Silamikelis I., Terentjeva A., Perkons I., Birzniece L., Rovite V., Rozentale B., Viksna L., Kolesova O. (2021). Amino Acid Metabolism is Significantly Altered at the Time of Admission in Hospital for Severe COVID-19 Patients: Findings from Longitudinal Targeted Metabolomics Analysis. Microbiol. Spectr..

[75] Masoodi M., Peschka M., Schmiedel S., Haddad M., Frye M., Maas C., Lohse A., Huber S., Kirchhof P., Nofer J.-R. (2022). Disturbed lipid and amino acid metabolisms in COVID-19 patients. J. Mol. Med..

[76] Suhail S., Zajac J., Fossum C., Lowater H., McCracken C., Severson N., Laatsch B., Narkiewicz-Jodko A., Johnson B., Liebau J. (2020). Role of Oxidative Stress on SARS-CoV (SARS) and SARS-CoV-2 (COVID-19) Infection: A Review. Protein J..

[77] Chernyak B.V., Popova E.N., Prikhodko A.S., Grebenchikov O.A., Zinovkina L.A., Zinovkin R.A. (2020). COVID-19 and Oxidative Stress. Biochemistry.

[78] Pincemail J., Cavalier E., Charlier C., Cheramy–Bien J.-P., Brevers E., Courtois A., Fadeur M., Meziane S., Goff C.L., Misset B. (2021). Oxidative Stress Status in COVID-19 Patients Hospitalized in Intensive Care Unit for Severe Pneumonia. A Pilot Study. Antioxidants.

[79] Dasari C.M., Bhukya R. (2020). Comparative analysis of protein synthesis rate in COVID-19 with other human coronaviruses. Infect. Genet. Evol..

[80] Yuan S., Peng L., Park J.J., Hu Y., Devarkar S.C., Dong M.B., Shen Q., Wu S., Chen S., Lomakin I.B. (2020). Nonstructural Protein 1 of SARS-CoV-2 Is a Potent Pathogenicity Factor Redirecting Host Protein Synthesis Machinery toward Viral RNA. Mol. Cell.

[81] Woods J.A., Hutchinson N.T., Powers S.K., Roberts W.O., Gomez-Cabrera M.C., Radak Z., Berkes I., Boros A., Boldogh I., Leeuwenburgh C. (2020). The COVID-19 pandemic and physical activity. Sport. Med. Health Sci..

[82] Altay O., Arif M., Li X., Yang H., Aydın M., Alkurt G., Kim W., Akyol D., Zhang C., Dinler-Doganay G. (2021). Combined Metabolic Activators Accelerates Recovery in Mild-to-Moderate COVID-19. Adv. Sci..

[83] Chuan-Yuan L. (2020). Can Glycine Mitigate COVID-19 Associated Tissue Damage and Cytokine Storm?. Radiat. Res..

[84] Silvagno F., Vernone A., Pescarmona G.P. (2020). The Role of Glutathione in Protecting against the Severe Inflammatory Response Triggered by COVID-19. Antioxidants.

[85] Bak D.W., Bechtel T.J., Falco J.A., Weerapana E. (2019). Cysteine reactivity across the subcellular universe. Curr. Opin. Chem. Biol..

[86] Bonvini A., Coqueiro A.Y., Tirapegui J., Calder P.C., Rogero M.M. (2018). Immunomodulatory role of branched-chain amino acids. Nutr. Rev..

[87] Tsai S.C., Lu C.C., Bau D.T., Chiu Y.J., Yen Y.T., Hsu Y.M., Fu C.W., Kuo S.C., Lo Y.S., Chiu H.Y. (2021). Approaches towards fighting the COVID-19 pandemic (Review). Int. J. Mol. Med..

[88] Volz E., Hill V., McCrone J.T., Price A., Jorgensen D., O’Toole Á., Southgate J., Johnson R., Jackson B., Nascimento F.F. (2021). Evaluating the Effects of SARS-CoV-2 Spike Mutation D614G on Transmissibility and Pathogenicity. Cell.

[89] Cervenka I., Agudelo L.Z., Ruas J.L. (2017). Kynurenines: Tryptophan’s metabolites in exercise, inflammation, and mental health. Science.

[90] Lawler N.G., Gray N., Kimhofer T., Boughton B., Gay M., Yang R., Morillon A.-C., Chin S.-T., Ryan M., Begum S. (2021). Systemic Perturbations in Amine and Kynurenine Metabolism Associated with Acute SARS-CoV-2 Infection and Inflammatory Cytokine Responses. J. Proteome Res..

[91] Oh M.-H., Sun I.-H., Zhao L., Leone R.D., Sun I.-M., Xu W., Collins S.L., Tam A.J., Blosser R.L., Patel C.H. (2020). Targeting glutamine metabolism enhances tumor-specific immunity by modulating suppressive myeloid cells. J. Clin. Investig..

[92] Kretzmann N.A., Fillmann H., Mauriz J.L., Marroni C.A., Marroni N., González-Gallego J., Tuñón M.J. (2008). Effects of glutamine on proinflammatory gene expression and activation of nuclear factor kappa B and signal transducers and activators of transcription in TNBS-induced colitis. Inflamm. Bowel Dis..

[93] Gómez-Mesa J.E., Galindo-Coral S., Montes M.C., Muñoz Martin A.J. (2021). Thrombosis and Coagulopathy in COVID-19. Curr. Probl. Cardiol..

[94] Watanabe K., Nagao M., Toh R., Irino Y., Shinohara M., Iino T., Yoshikawa S., Tanaka H., Satomi-Kobayashi S., Ishida T. (2021). Critical role of glutamine metabolism in cardiomyocytes under oxidative stress. Biochem. Biophys. Res. Commun..

[95] Mohajeri M., Horriatkhah E., Mohajery R. (2021). The effect of glutamine supplementation on serum levels of some inflammatory factors, oxidative stress, and appetite in COVID-19 patients: A case-control study. Inflammopharmacology.

[96] Kerner J., Hoppel C.L., Lennarz W.J., Lane M.D. (2013). Carnitine and β-Oxidation. Encyclopedia of Biological Chemistry.

[97] Houten S.M., Wanders R.J.A., Ranea-Robles P. (2020). Metabolic interactions between peroxisomes and mitochondria with a special focus on acylcarnitine metabolism. Biochim. Biophys. Acta Mol. Basis Dis..

[98] Boenzi S., Diodato D. (2018). Biomarkers for mitochondrial energy metabolism diseases. Essays. Biochem..

[99] Indiveri C., Iacobazzi V., Tonazzi A., Giangregorio N., Infantino V., Convertini P., Console L., Palmieri F. (2011). The mitochondrial carnitine/acylcarnitine carrier: Function, structure and physiopathology. Mol. Asp. Med..

[100] Kerner J., Hoppel C. (2000). Fatty acid import into mitochondria. Biochim. Biophys. Acta.

[101] Chen W.S., Liu M.H., Cheng M.L., Wang C.H. (2020). Decreases in Circulating Concentrations of Short-Chain Acylcarnitines are Associated with Systolic Function Improvement After Decompensated Heart Failure. Int. Heart J..

[102] Li X., Li Y., Liang Y., Hu R., Xu W., Liu Y. (2021). Plasma Targeted Metabolomics Analysis for Amino Acids and Acylcarnitines in Patients with Prediabetes, Type 2 Diabetes Mellitus, and Diabetic Vascular Complications. Diabetes Metab. J..

[103] Korobkova E.O., Kozhevnikova M.V., Ilgisonis I.S., Shakaryants G.A., Appolonova S.A., Kukharenko A.V., Larcova E.V., Maltseva A.A., Khabarova N.V., Belenkov Y.N. (2020). Metabolomic profiling in patients with metabolic syndrome. Kardiologiia.

[104] Ejaz H., Alsrhani A., Zafar A., Javed H., Junaid K., Abdalla A.E., Abosalif K.O.A., Ahmed Z., Younas S. (2020). COVID-19 and comorbidities: Deleterious impact on infected patients. J. Infect. Public Health.

[105] Tajbakhsh A., Gheibi Hayat S.M., Taghizadeh H., Akbari A., Inabadi M., Savardashtaki A., Johnston T.P., Sahebkar A. (2021). COVID-19 and cardiac injury: Clinical manifestations, biomarkers, mechanisms, diagnosis, treatment, and follow up. Expert Rev. Anti. Infect..

[106] Wang T., Du Z., Zhu F., Cao Z., An Y., Gao Y., Jiang B. (2020). Comorbidities and multi-organ injuries in the treatment of COVID-19. Lancet.

[107] Huang C., Huang L., Wang Y., Li X., Ren L., Gu X., Kang L., Guo L., Liu M., Zhou X. (2021). 6-month consequences of COVID-19 in patients discharged from hospital: A cohort study. Lancet.

[108] Otsubo C., Bharathi S., Uppala R., Ilkayeva O.R., Wang D., McHugh K., Zou Y., Wang J., Alcorn J.F., Zuo Y.Y. (2015). Long-chain Acylcarnitines Reduce Lung Function by Inhibiting Pulmonary Surfactant. J. Biol. Chem..

