# Supplementary material for: Urine Metabolites Enable Fast Detection of COVID-19 Using Mass Spectrometry
Source: Metabolites. 2022 Nov 2;12(11):1056. doi: 10.3390/metabo12111056 (PMC9697918; doi:10.3390/metabo12111056)
Supplement: Supplementary file 1 [file metabolites-12-01056-s001.zip › metabolites-1908649-supplementary.pdf]

URINE METABOLITES ENABLE FAST DETECTION OF COVID-19 USING MASS SPECTROMETRY

Alexandre Varao Moura[1†], Danilo Cardoso de Oliveira[1†], Alex Ap. R. Silva[1], Jonas Ribeiro da Rosa[1], Pedro Henrique Dias Garcia[1], Pedro Henrique Godoy Sanches[1], Kyana Y. Garza[2], Flavio Marcio Macedo Mendes [1], Mayara Lambert[1], Junier Marrero Gutierrez[1], Nicole Marino Granado[1], Alicia Camacho dos Santos[5], Iasmin Lopes de Lima[5], Lisamara Dias de Oliveira Negrini[3], Marcia Aparecida Antonio[4], Marcos N. Eberlin[5], Livia S. Eberlin[2,6,] and Andreia de Melo Porcari[1*]

1 MS[4]Life Laboratory of Mass Spectrometry, Health Sciences Postgraduate Program, São Francisco University, Bragança Paulista, São Paulo, Brazil.
2 Department of Chemistry, The University of Texas at Austin, Austin, Texas 78712, United States.
3 Municipal Department of Health, Bragança Paulista, Sao Paulo, Brazil
4 Integrated Unit of Pharmacology and Gastroenterology, UNIFAG, Bragança Paulista, Sao Paulo, Brazil
5 Department of Material Engineering and Nanotechnology, Mackenzie Presbyterian University, São Paulo, Sao Paulo, Brazil
6 Department of Surgery, Baylor College of Medicine, Houston, Texas 77030, United States.
* Correspondence: andreia.porcari@usf.edu.br; Tel.: +55-11-2454-8047
† These authors contributed equally

LIST OF CONTENTS:

- **Supporting Figures**
  - **Supporting Figure S1.** Metabolite Enrichment Analysis for the discriminatory analytes found by Lasso.
  - **Supporting Figure S2.** Metabolite Enrichment Analysis by Over Representation Analysis for the discriminatory analytes found by Lasso.
- **Supporting Tables**
  - **Supplementary Table S1:** Amino Acids, Acylcarnitines investigated in urine from COVID patients and their experimental detection parameters.
  - **Supplementary Table S2:** Relative Standard Deviation of metabolites and internal standard for QC samples during batch analysis and after the heat inactivation
  - **Supplementary Table S3:** Aminoacids and Acylcarntines selected after RSD and IQR filtering processes performed in MetaboAnalyst
  - **Supplementary Table S4:** The effect of age and sex on the model's performance.
  - **Supplementary Table S5A:** Comparison of analytes between the groups (Pos-H and Neg-NH) of patients using the Mann-Whitney test.
  - **Supplementary Table S5B:** Comparison analytes between the groups (Pos-H, Neg-NH, and Neg-H) of patients using the Kruskal-Wallis test and Dun's Test as pos-hoc.
  - **Supplementary Table S6A:** Classification of the Withheld Set 1 patients and their clinical characteristics.
  - **Supplementary Table S6B:** Classification of the Withheld Set 2 patients and their clinical characteristics.

SUPPORTING FIGURES

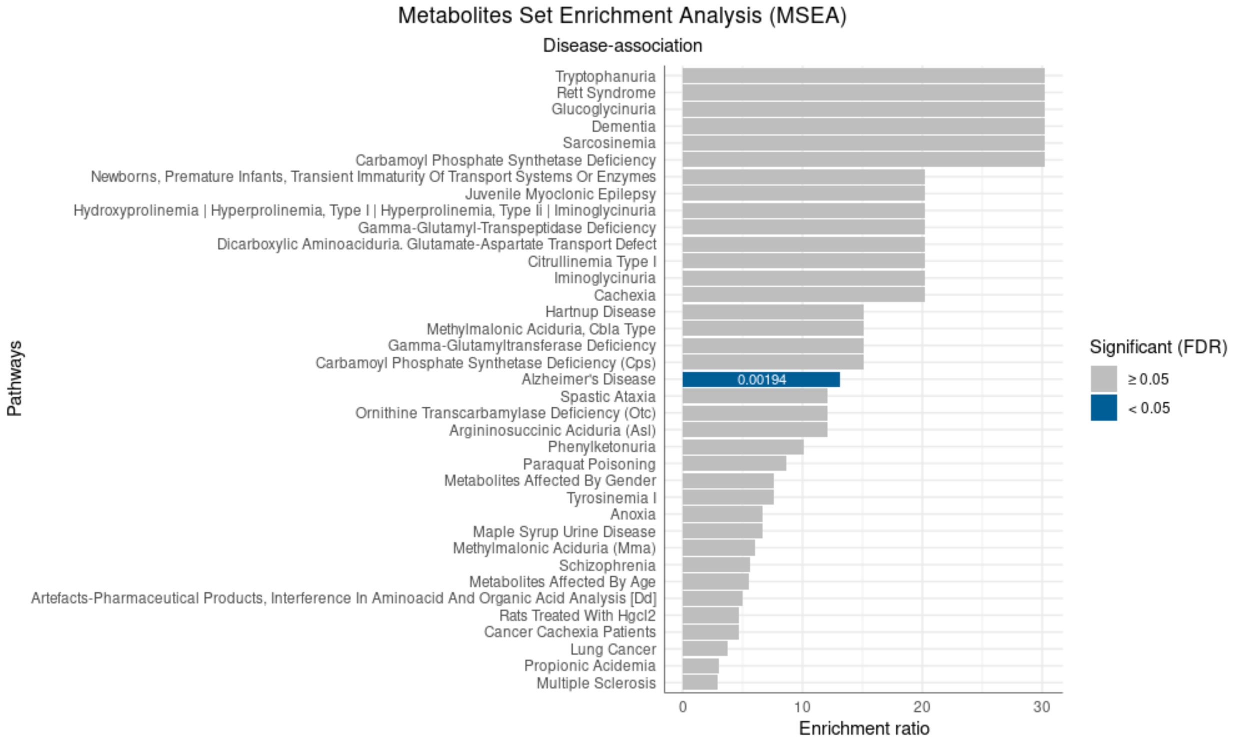

Supplementary Figure S1. Metabolite Enrichment Analysis for the discriminatory analytes found by Lasso. A set of disease-associated metabolites from human urine was used, and the p-adjusted values (FDR) for associated diseases are presented by the color bar, with the significant ones (FDR < 0.05) displayed numerically. The x-axis represent the Enrichment Ratio (*hits/expected hit*) from the particular disease.

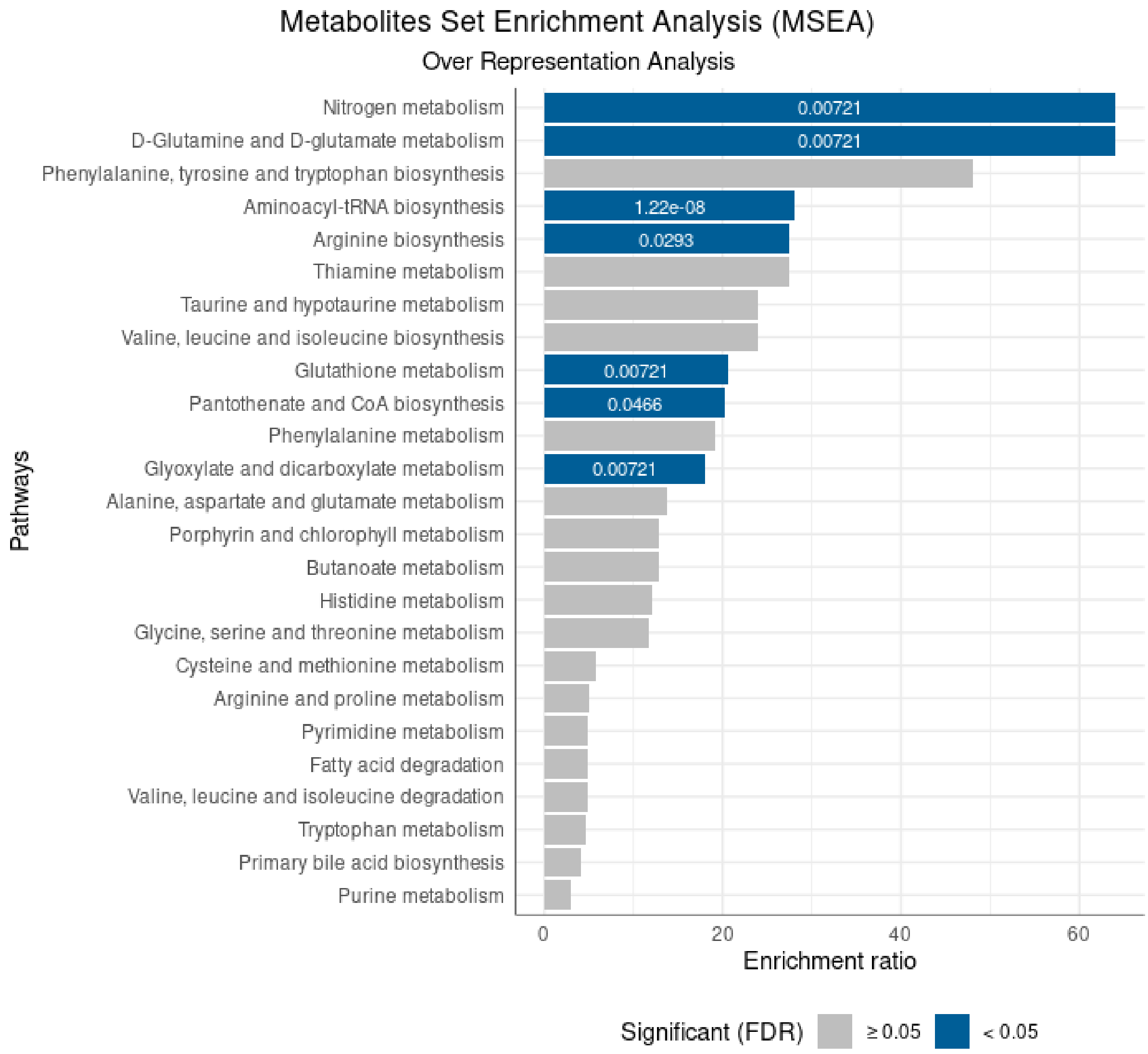

Supplementary Figure S2. Metabolite Enrichment Analysis by Over Representation Analysis for the discriminatory analytes found by Lasso. A set of human metabolites from KEGG library was used, and the p-adjusted values for the pathways are presented by the color bar, with the significant ones (FDR < 0.05) displayed numerically.

SUPLEMENTARY TABLES

Supplementary Table S1: Amino Acids, Acylcarnitines investigated in urine from COVID patients and their experimental detection parameters.

**Aminoacids (19)**			
Compound	***Cone (V)***	***Collision (eV)***	***MRM (Transition, m/z)***
Alanine	20	8	90.1 - 44.1
Arginine	30	21	175.1 - 70.1
Asparagine	20	6	133.1 - 87.0
Aspartate	14	10	134.1 - 88.0
Citrulline	20	14	176.0 - 113.0
Cysteine	20	10	122.1 - 105.0
Glutamic Acid	20	8	148.1 - 130.0
Glutamine	20	8	130.0 - 84.1
Glycine	20	7	76.0 - 30.1
Histidine	20	16	156.1 - 110.0
Leucine	20	9	132.1 - 85.1
Lysine	14	14	147.1 - 84.0
Phenylalanine	20	12	166.1 - 120.1
Proline	20	10	116.1 - 70.0
Serine	14	8	106.1 - 60.0
Threonine	38	20	120.1 - 74.0
Tryptophan	16	18	205.1 - 146.0
Tyrosine	20	12	182.1 - 136.1
Valine	20	10	118.1 - 72.1
**AcylCarnitines (15)**			
Compound	***Cone (V)***	***Collision (eV)***	***MRM (Transition. m/z)***
Octadecanoyl - Carnitine (C18)	40	19	428.0 - 85.0
Free Carnitine (C0)	40	19	162.0 - 85.0
Decanoyl - Carnitine (C10)	40	19	316.0 - 85.0
Dodecanoyl - Carnitine (C12)	40	19	344.0 - 85.0
Tetradecanoyl - Carnitine (C14)	40	28	372.0 - 85.0
Palmitoyl - Carnitine (C16)	40	25	400.3 - 85.0
Hydroxypalmitoyl - Carnitine (C16OH)	40	25	416.0-85.0
Octadecenoyl - Carnitine (C18:1)	40	19	426.0 - 85.0
Acetyl - Carnitine (C2)	35	19	204.1 - 85.0
Propionyl - Carnitine (C3)	35	19	218.1 - 85.0
Butyryl - Carnitine (C4)	35	19	232.1 - 85.0
Isovaleryl - Carnitine (C5)	35	19	246.1 - 85.0
Hexanoyl - Carnitine (C6)	35	19	260.0 - 85.0
Octanoyl - Carnitine (C8)	40	19	288.1 - 85.0
Malonyl - Carnitine (C3DC)	40	19	248.0 - 85.0
Isovaleryl - Carnitine (C5D9)*	40	19	255.1 - 85.0

* used as internal standard

Supplementary Table S2: Relative Standard Deviation of metabolites and internal standard for QC samples during batch analysis and after the heat inactivation

**Metabolites**	**RSD**<br>**QC (N = 28)**	**RSD**<br>**Heat inactivation* (N=12)**
Aspartate	61.3	32.6
Asparagine	34.9	4.9
Proline	25.0	12.2
C-18	24.2	4.7
Ac Glutamic	19.2	2.8
C18-1	18.5	4.2
Citrulline	12.2	13.3
Alanine	10.6	4.2
Valine	10.3	3.7
C16OH	9.8	9.9
Cysteine	9.3	1.6
Threonine	8.8	1.2
Glutamine	8.2	2.1
C16	7.7	3.3
C14	7.4	1.7
Leucine	6.9	3.9
Glycine	6.2	2.2
Arginine	5.8	2.4
Serine	5.8	4.0
Tyrosine	5.8	4.3
Tryptophan	5.8	2.2
Lysine	5.5	1.6
Histidine	5.5	3.2
Phenylalanine	5.4	2.5
C12	5.2	1.2
Malonil.C3DC	5.0	1.0
C6	4.7	1.9
C2	4.7	4.0
C3	4.2	3.2
C0	4.1	2.0
C8	3.6	4.1
C10	3.4	1.0
C5	3.4	1.4
C4	3.4	1.3
C5D9**	3.3	

* prior and after heat innactivation

** internal standard

Supplementary Table S3: Aminoacids and Acylcarntines selected after RSD and IQR filtering processes performed in MetaboAnalyst

**Aminoacids (16)**
Alanine
Leucine
Glycine
Lysine
Threonine
Glutamic Acid
Cysteine
Glutamine
Phenylalanine
Tryptophan
Arginine
Citrulline
Histidine
Serine
Valine
Tyrosine
**AcylCarnitines (13)**
C0
C5
C12
C10
C2
C6
C14
C16OH
C3
C8
C16
Malonil C3DC
C4

Supplementary Table S4: The effect of age and sex on the model's performance[1].

		Original Classifier	Age and Sex corrected Classifier[a]	Age and Sex corrected Classifier[b]
Variable	(Intercept)	-0.46	-1.20	-0.60
	Glycine	-3.81	-3.67	-3.67
	Valine	-2.85	-2.71	-2.83
	C8	-1.68	-1.68	-1.70
	C6	-1.16	-1.23	-1.14
	C16	-0.50	-0.55	-0.58
	Cysteine	-0.48	-0.48	-0.48
	C2	-0.09	not listed	-0.07
	Age	not listed	0.02	0.21
	C12	0.10	0.23	0.20
	Tryptophan	0.35	0.43	0.33
	Phenylalanine	0.53	0.43	0.52
	Glutamine	0.59	0.46	0.56
	C5	0.71	0.76	0.73
	Glutamic Acid	0.94	0.95	0.94
	C16OH	1.73	1.61	1.66
Performance	**Training Set**			
	Sens (%)	97.00	100.00	94.00
	Spec (%)	95.00	94.00	97.00
	PPV (%)	89.00	86.00	94.00
	NPV (%)	99.00	100.00	97.00
	Acc. (%)	95.00	95.00	96.00
	CI (95%)	0.90-0.99	0.90-0.99	0.91-0.99
	**Test Set**			
	Sens. (%)	90.00	100.00	90.00
	Spec. (%)	100.00	100.00	100.00
	PPV (%)	100.00	100.00	100.00
	NPV (%)	96.00	100.00	96.00
	Acc. (%)	97.00	100.00	97.00
	CI (95%)	0.85-1.00	0.90-1.00	0.90-1.00

[a] age considered as a numeric variable; [b] age considered as a categorical variable, being ≤ 38 (group 0), and > 38 (group 1). Sex was considered a categorical variable for both models.

[1] To verify the influence of age and sex on the classification performance of our model, we generated two additional models. The first one includes age as a categorical variable, and the second includes age as a numerical variable. Both included sex as a categorical variable. The results are compared in the ST4. Although age was ranked as relevant by Lasso in both new models, it presented low coefficients. The use of age slightly improved the classification performance results for both additional models. However, we opted not to take this variable into account in the manuscript and to keep the independence of the model concerning age, as we believe this model can be adapted to different populations in the future. Additionally the distribution of individuals is similar to the incidence of the disease in Brazil by the time recruitment was done (Boletim Epidemiológico N° 36. Ministério da Saúde, 2020). There was a higher incidence at the average age of 59 years old, which is close to the observed age in the Pos-H group.

Supplementary Table S5A: Comparison of analytes between the groups (Pos-H and Neg-NH) of patients using the Mann-Whitney test.

Compound	POS_H	NEG_NH	fold.change	p.value	p.adjust	p.adjust
Alanine	5.83	15.46	-1.41	1.10E-10	5.33E-10	5E-10
Glycine	4.62	23.31	-2.33	8.64E-19	2.51E-17	3E-17
Cysteine	2.07	13.16	-2.67	9.97E-13	9.64E-12	1E-11
Threonine	0.60	0.91	-0.60	2.17E-04	3.50E-04	3E-04
Serine	6.05	10.66	-0.82	4.16E-08	1.34E-07	1E-07
Glutamine	5.21	7.57	-0.54	2.30E-01	2.47E-01	2E-01
Leucine	19.08	34.28	-0.85	1.88E-06	4.19E-06	4E-06
Valine	6.02	21.73	-1.85	1.14E-16	1.65E-15	2E-15
Lysine	49.30	76.16	-0.63	1.93E-05	3.72E-05	4E-05
Glutamic.Acid	2.19	2.73	-0.31	3.52E-01	3.64E-01	4E-01
Histidine	137.40	269.51	-0.97	4.15E-12	3.01E-11	3E-11
C0	161.36	57.97	1.48	1.39E-06	3.66E-06	4E-06
Phenylalanine	23.11	36.65	-0.67	8.58E-05	1.56E-04	2E-04
C4	60.72	77.75	-0.36	3.09E-04	4.48E-04	4E-04
Arginine	7.61	13.99	-0.88	6.19E-06	1.28E-05	1E-05
Citrulline	0.40	0.60	-0.60	2.80E-04	4.27E-04	4E-04
Tyrosine	7.93	10.21	-0.36	8.63E-03	1.04E-02	1E-02
C2	332.37	154.02	1.11	1.51E-06	3.66E-06	4E-06
Tryptophan	7.48	10.50	-0.49	1.86E-04	3.17E-04	3E-04
C3	42.16	13.19	1.68	7.60E-11	4.41E-10	4E-10
C5	50.97	37.36	0.45	7.76E-04	1.02E-03	1E-03
Malonil.C3DC	8.61	4.25	1.02	3.21E-09	1.33E-08	1E-08
C6	4.40	7.97	-0.86	1.59E-07	4.61E-07	5E-07
C8	9.51	12.67	-0.41	9.50E-04	1.20E-03	1E-03
C10	7.01	10.91	-0.64	3.59E-04	4.95E-04	5E-04
C12	3.40	4.36	-0.36	5.58E-02	6.22E-02	6E-02
C14	1.02	1.62	-0.67	9.92E-03	1.15E-02	1E-02
C16	0.56	1.21	-1.12	2.19E-08	7.94E-08	8E-08
C16OH	0.54	0.58	-0.10	9.84E-01	9.84E-01	1E+00

The correction of p-value was performed with Benjamini-Hochberg (BH).

Supplementary Table S5B: Comparison analytes between the groups (Pos-H, Neg-NH, and Neg-H) of patients using the Kruskal-Wallis test and Dun's Test as pos-hoc.

Compound	Label1	p-value (Dun's test)	p-adj	Label2	p-value (Dun's test)	p-adj	Label3	p-value (Dun's test)	p-adj	p-value (Kruskal-Wallis test)
Alanine	NEG_NH - POS_H	5.26E-11	1.58E-10	NEG_H - POS_H	8.78E-01	8.78E-01	NEG_H - NEG_NH	4.44E-08	6.66E-08	1.17E-13
Glycine	NEG_NH - POS_H	1.07E-18	3.21E-18	NEG_H - POS_H	1.41E-01	1.41E-01	NEG_H - NEG_NH	4.69E-08	7.03E-08	2.54E-20
Cysteine	NEG_NH - POS_H	5.65E-13	1.69E-12	NEG_H - POS_H	9.43E-01	9.43E-01	NEG_H - NEG_NH	9.48E-09	1.42E-08	8.36E-16
Threonine	NEG_NH - POS_H	2.12E-04	3.18E-04	NEG_H - POS_H	1.66E-01	1.66E-01	NEG_H - NEG_NH	5.22E-06	1.57E-05	7.69E-07
Serine	NEG_NH - POS_H	3.72E-08	1.12E-07	NEG_H - POS_H	6.64E-01	6.64E-01	NEG_H - NEG_NH	8.03E-07	1.20E-06	2.26E-10
Glutamine	NEG_NH - POS_H	1.66E-01	1.66E-01	NEG_H - POS_H	3.78E-02	5.67E-02	NEG_H - NEG_NH	5.32E-04	1.60E-03	2.07E-03
Leucine	NEG_NH - POS_H	2.16E-06	6.48E-06	NEG_H - POS_H	5.61E-01	5.61E-01	NEG_H - NEG_NH	7.38E-06	1.11E-05	3.16E-08
Valine	NEG_NH - POS_H	1.18E-16	3.53E-16	NEG_H - POS_H	9.64E-01	9.64E-01	NEG_H - NEG_NH	3.17E-11	4.76E-11	9.83E-21
Lysine	NEG_NH - POS_H	1.57E-05	2.35E-05	NEG_H - POS_H	2.63E-01	2.63E-01	NEG_H - NEG_NH	2.03E-06	6.08E-06	5.32E-08
Glutamic.Acid	NEG_NH - POS_H	3.29E-01	9.88E-01	NEG_H - POS_H	9.41E-01	9.41E-01	NEG_H - NEG_NH	3.84E-01	5.76E-01	4.99E-01
Histidine	NEG_NH - POS_H	1.02E-11	3.07E-11	NEG_H - POS_H	7.69E-01	7.69E-01	NEG_H - NEG_NH	5.75E-09	8.62E-09	6.54E-15
C0	NEG_NH - POS_H	3.01E-07	9.02E-07	NEG_H - POS_H	7.84E-01	7.84E-01	NEG_H - NEG_NH	8.76E-06	1.31E-05	7.37E-09
Phenylalanine	NEG_NH - POS_H	1.05E-04	1.58E-04	NEG_H - POS_H	1.42E-01	1.42E-01	NEG_H - NEG_NH	1.65E-06	4.95E-06	1.81E-07
C4	NEG_NH - POS_H	3.65E-04	1.10E-03	NEG_H - POS_H	5.38E-01	5.38E-01	NEG_H - NEG_NH	2.92E-02	4.37E-02	6.63E-04
Arginine	NEG_NH - POS_H	4.01E-06	6.02E-06	NEG_H - POS_H	3.02E-01	3.02E-01	NEG_H - NEG_NH	1.02E-06	3.05E-06	1.09E-08

Compound	Label1	p-value (Dun's test)	p-adj	Label2	p-value (Dun's test)	p-adj	Label3	p-value (Dun's test)	p-adj	p-value (Kruskal-Wallis test)
Citrulline	NEG_NH - POS_H	2.58E-04	3.86E-04	NEG_H - POS_H	4.95E-01	4.95E-01	NEG_H - NEG_NH	1.98E-04	5.94E-04	1.60E-05
Tyrosine	NEG_NH - POS_H	8.42E-03	1.26E-02	NEG_H - POS_H	1.84E-01	1.84E-01	NEG_H - NEG_NH	2.85E-04	8.55E-04	2.53E-04
C2	NEG_NH - POS_H	1.89E-06	2.83E-06	NEG_H - POS_H	8.97E-02	8.97E-02	NEG_H - NEG_NH	8.21E-09	2.46E-08	1.23E-10
Tryptophan	NEG_NH - POS_H	2.22E-04	6.65E-04	NEG_H - POS_H	6.22E-01	6.22E-01	NEG_H - NEG_NH	4.02E-04	6.02E-04	2.45E-05
C3	NEG_NH - POS_H	3.14E-11	9.43E-11	NEG_H - POS_H	3.34E-01	3.34E-01	NEG_H - NEG_NH	1.10E-10	1.66E-10	7.83E-16
C5	NEG_NH - POS_H	6.56E-04	1.97E-03	NEG_H - POS_H	7.87E-01	7.87E-01	NEG_H - NEG_NH	1.45E-02	2.17E-02	7.09E-04
Malonil.C3DC	NEG_NH - POS_H	2.75E-09	8.24E-09	NEG_H - POS_H	6.71E-01	6.71E-01	NEG_H - NEG_NH	1.28E-07	1.93E-07	6.78E-12
C6	NEG_NH - POS_H	6.52E-08	9.78E-08	NEG_H - POS_H	2.64E-01	2.64E-01	NEG_H - NEG_NH	1.85E-08	5.56E-08	1.88E-11
C8	NEG_NH - POS_H	7.32E-04	1.10E-03	NEG_H - POS_H	3.64E-01	3.64E-01	NEG_H - NEG_NH	1.75E-04	5.24E-04	3.17E-05
C10	NEG_NH - POS_H	2.10E-04	3.15E-04	NEG_H - POS_H	2.33E-01	2.33E-01	NEG_H - NEG_NH	1.43E-05	4.30E-05	1.74E-06
C12	NEG_NH - POS_H	4.79E-02	7.18E-02	NEG_H - POS_H	3.32E-01	3.32E-01	NEG_H - NEG_NH	7.09E-03	2.13E-02	1.01E-02
C14	NEG_NH - POS_H	7.64E-03	1.15E-02	NEG_H - POS_H	5.14E-01	5.14E-01	NEG_H - NEG_NH	3.83E-03	1.15E-02	1.86E-03
C16	NEG_NH - POS_H	6.48E-09	1.94E-08	NEG_H - POS_H	7.67E-01	7.67E-01	NEG_H - NEG_NH	1.35E-05	2.03E-05	4.12E-10
C16OH	NEG_NH - POS_H	9.74E-01	9.74E-01	NEG_H - POS_H	4.91E-01	7.37E-01	NEG_H - NEG_NH	4.21E-01	1.00E+00	7.15E-01

The correction of p-value was performed with Benjamini-Hochberg (BH).

Supplementary Table S6A: Classification of the Withheld Set 1 patients and their clinical characteristics.

**Patient ID #**	**30**	**34**	**36**	**45**	**68**	**71**	**92**
**Clinical Classification**	NEG_H	NEG_H	NEG_H	NEG_H	NEG_H	NEG_H	NEG_H
**Model Prediction Result**	NEG_NH	POS_H	POS_H	POS_H	POS_H	NEG_NH	POS_H
Gender	F	M	M	F	F	F	F
Age	35	76	73	50	76	49	69
RT-PCR results	NEG	NEG	NEG	NEG	NEG	NEG	NEG
Days.in.between.PCR.and.MS.sample.collection	2	1	2	1	2	2	1
MS.Collection.Symptoms.onset.difference	-13	-8	-4	-14	-3	-5	-11
Mechanical.ventilation..days.	0	11	0	0	0	0	19
Fever	YES	NO	YES	NO	YES	YES	NO
Cough	YES	YES	NO	NO	NO	NO	YES
Myalgia	NO	NO	NO	YES	NO	NO	NO
Sore.Throat	NO	YES	NO	NO	NO	YES	NO
Headache	NO	NO	NO	NO	NO	NO	NO
Coryza	NO	NO	NO	NO	NO	NO	NO
Dyspnea	YES	YES	YES	YES	YES	NO	YES
SatO2.95	YES	NO	YES	NO	YES	YES	NO
Tiredness.fatigue.	NO	NO	NO	NO	NO	NO	NO
Loss.of.smell.or.taste	YES	NO	NO	NO	YES	YES	NO
Vomits.or.nausea	NO	NO	NO	NO	YES	NO	NO
Diarrhea	YES	NO	NO	NO	NO	NO	NO
SAH..Systemic.Arterial.Hypertension.	NO	YES	YES	YES	NO	NO	YES
Cardiovascular.disease..not.SAH.	NO	NO	NO	NO	NO	NO	NO
Obesity	NO	NO	NO	NO	NO	NO	NO
DM..Diabetes.Melitus.	NO	NO	NO	NO	NO	NO	NO
Neoplasias	NO	NO	NO	NO	NO	NO	NO
Lung.Disease	NO	NO	YES	NO	NO	NO	NO
COPD..Chronic.Obstrutive.Pulmonar.Disease.	NO	NO	NO	NO	NO	NO	NO
Smoker.or.Ex.Smoker.	NO	NO	NO	NO	NO	NO	NO
Asthma	NO	NO	NO	NO	NO	NO	NO
Kidney.disease	NO	NO	NO	NO	NO	NO	NO
Ground.glass.Opacity	YES	YES	YES	YES	NO	YES	YES
Pulmonary.Commitment.Degree	YES	YES	YES	YES	NO	YES	YES
Suggestive.of.viral.infection	YES	YES	YES	YES	NO	YES	YES

Supplementary Table S6A: Classification of the Withheld Set 1 patients and their clinical characteristics.

**Patient ID #**	**93**	**96**	**97**	**101**	**108**	**111**	**116**
**Clinical Classification**	NEG_H	NEG_H	NEG_H	NEG_H	NEG_H	NEG_H	NEG_H
**Model Prediction Result**	POS_H	POS_H	POS_H	POS_H	POS_H	POS_H	POS_H
Gender	M	M	M	F	F	M	F
Age	68	76	67	27	60	50	78
RT-PCR results	NEG	NEG	NEG	NEG	NEG	NEG	NEG
Days.in.between.PCR.and.MS.sample.collection	2	2	2	0	1	2	2
MS.Collection.Symptoms.onset.difference	-10	-6	-7	-8	-11	-11	-13
Mechanical.ventilation..days.	0	0	0	0	0	0	0
Fever	NO	NO	YES	YES	NO	NO	NO
Cough	YES	YES	NO	NO	YES	YES	NO
Myalgia	NO	NO	NO	NO	NO	NO	NO
Sore.Throat	NO	NO	YES	NO	NO	NO	NO
Headache	NO	NO	NO	NO	NO	NO	NO
Coryza	NO	NO	YES	NO	NO	NO	NO
Dyspnea	YES	YES	NO	NO	NO	NO	YES
SatO2.95	NO	NO	NO	YES	YES	YES	NO
Tiredness.fatigue.	NO	NO	NO	NO	YES	NO	NO
Loss.of.smell.or.taste	YES	NO	NO	NO	NO	YES	YES
Vomits.or.nausea	NO	NO	NO	YES	NO	YES	NO
Diarrhea	NO	NO	NO	NO	NO	NO	YES
SAH..Systemic.Arterial.Hypertension.	NO	NO	YES	NO	YES	NO	NO
Cardiovascular.disease..not.SAH.	NO	YES	NO	NO	NO	NO	YES
Obesity	NO	NO	NO	NO	YES	NO	NO
DM..Diabetes.Melitus.	NO	YES	NO	NO	NO	NO	NO
Neoplasias	NO	NO	NO	NO	NO	NO	NO
Lung.Disease	NO	YES	NO	YES	NO	NO	NO
COPD..Chronic.Obstrutive.Pulmonar.Disease.	NO	YES	NO	NO	NO	NO	NO
Smoker.or.Ex.Smoker.	NO	YES	NO	NO	NO	NO	NO
Asthma	NO	NO	NO	YES	NO	NO	NO
Kidney.disease	NO	NO	NO	NO	NO	NO	NO
Ground.glass.Opacity	YES	YES	YES	YES	YES	YES	
Pulmonary.Commitment.Degree	YES	YES	YES	NO	YES	YES	
Suggestive.of.viral.infection	YES	YES	YES	YES	YES	YES	NO

Supplementary Table S6A: Classification of the Withheld Set 1 patients and their clinical characteristics.

**Patient ID #**	**149**	**151**	**152**	**156**	**193**	**240**	**242**
**Clinical Classification**	NEG_H	NEG_H	NEG_H	NEG_H	NEG_H	NEG_H	NEG_H
**Model Prediction Result**	POS_H	POS_H	POS_H	POS_H	POS_H	POS_H	POS_H
Gender	M	M	M	F	F	M	M
Age	66	47	66	81	57	68	28
RT-PCR results	NEG	NEG	NEG	NEG	NEG	NEG	NEG
Days.in.between.PCR.and.MS.sample.collection	0	1	1	2	-1	1	2
MS.Collection.Symptoms.onset.difference	-11	-5	-5	-10	-11	-5	-9
Mechanical.ventilation..days.	0	0	0	0	0	0	0
Fever	YES	NO	YES	NO	YES	YES	NO
Cough	NO	YES	NO	YES	NO	YES	YES
Myalgia	NO	NO	NO	NO	YES	NO	NO
Sore.Throat	YES	YES	NO	YES	NO	NO	YES
Headache	NO	YES	NO	NO	NO	NO	NO
Coryza	NO	NO	NO	NO	NO	YES	YES
Dyspnea	NO	YES	YES	YES	NO	YES	NO
SatO2.95	NO	YES	NO	NO	YES	NO	NO
Tiredness.fatigue.	YES	NO	NO	NO	NO	NO	NO
Loss.of.smell.or.taste	NO	YES	YES	NO	YES	NO	NO
Vomits.or.nausea	NO	NO	NO	NO	NO	NO	NO
Diarrhea	NO	NO	NO	NO	NO	NO	NO
SAH..Systemic.Arterial.Hypertension.	NO	NO	NO	YES	YES	YES	YES
Cardiovascular.disease..not.SAH.	NO	NO	NO	YES	YES	NO	NO
Obesity	NO	NO	NO	NO	NO	NO	NO
DM..Diabetes.Melitus.	NO	NO	NO	NO	YES	NO	NO
Neoplasias	NO	NO	NO	NO	NO	NO	NO
Lung.Disease	NO	NO	NO	YES	NO	YES	NO
COPD..Chronic.Obstrutive.Pulmonar.Disease.	NO	NO	NO	NO	NO	NO	NO
Smoker.or.Ex.Smoker.	NO	NO	YES	NO	NO	YES	NO
Asthma	NO	NO	NO	YES	NO	NO	NO
Kidney.disease	NO	NO	NO	NO	NO	NO	NO
Ground.glass.Opacity	YES	YES	YES	NO	YES	NO	NO
Pulmonary.Commitment.Degree	YES	YES	YES	NO	YES	NO	NO
Suggestive.of.viral.infection	YES	YES	YES	NO	YES	NO	NO

Supplementary Table S6A: Classification of the Withheld Set 1 patients and their clinical characteristics.

**Patient ID #**	**264**	**267**	**270**
**Clinical Classification**	NEG_H	NEG_H	NEG_H
**Model Prediction Result**	POS_H	POS_H	POS_H
Gender	M	F	M
Age	26	52	67
RT-PCR results	NEG	NEG	NEG
Days.in.between.PCR.and.MS.sample.collection	2	-1	1
MS.Collection.Symptoms.onset.difference	-4	-2	-4
Mechanical.ventilation..days.	0	0	0
Fever	YES	NO	NO
Cough	YES	YES	YES
Myalgia	NO	NO	NO
Sore.Throat	YES	NO	NO
Headache	NO	NO	YES
Coryza	NO	NO	NO
Dyspnea	YES	YES	YES
SatO2.95	NO	NO	YES
Tiredness.fatigue.	NO	NO	NO
Loss.of.smell.or.taste	NO	NO	NO
Vomits.or.nausea	NO	NO	NO
Diarrhea	NO	NO	NO
SAH..Systemic.Arterial.Hypertension.	NO	NO	NO
Cardiovascular.disease..not.SAH.	NO	NO	NO
Obesity	NO	NO	NO
DM..Diabetes.Melitus.	NO	NO	NO
Neoplasias	NO	NO	NO
Lung.Disease	NO	NO	YES
COPD..Chronic.Obstrutive.Pulmonar.Disease.	NO	NO	YES
Smoker.or.Ex.Smoker.	NO	NO	YES
Asthma	NO	NO	NO
Kidney.disease	NO	NO	NO
Ground.glass.Opacity	YES	YES	YES
Pulmonary.Commitment.Degree	NO	YES	NO
Suggestive.of.viral.infection	YES	YES	YES

Supplementary Table S6B: Classification of the Withheld Set 2 patients and their clinical characteristics.

**Patient ID #**	**55**	**12**	**105**	**239**	**271**	**13**	**114**
**Clinical Classification**	NEG_H	NEG_H	NEG_H	NEG_H	NEG_H	NEG_H	NEG_H
**Model Prediction Result**	POS_H	NEG_NH	NEG_NH	POS_H	POS_H	POS_H	POS_H
Gender	M	F	F	M	F	F	M
Age	83	39	62	76	82	49	59
RT.PCR	NEG	NEG	NEG	NEG	NEG	NEG	NEG
Days.in.between.PCR.and.MS.sample.collection	3	1	4	3	5	6	95
MS.Collection.Symptoms.onset.difference	-6	-16	-10	-11	-12	-23	-23
Mechanical.ventilation..days.	0	0	0	0	0	0	0
Fever	NO	NO	YES	NO	YES	YES	NO
Cough	YES	YES	NO	YES	YES	YES	YES
Myalgia	NO	NO	NO	NO	NO	NO	NO
Sore.Throat	NO	NO	NO	NO	NO	NO	NO
Headache	NO	NO	NO	YES	NO	YES	NO
Coryza	NO	NO	NO	NO	NO	NO	NO
Dyspnea	YES	YES	NO	YES	YES	YES	YES
SatO2.95	NO	NO	NO	NO	NO	NO	NO
Tiredness.fatigue.	NO	NO	NO	NO	NO	NO	YES
Loss.of.smell.or.taste	NO	YES	NO	NO	NO	NO	YES
Vomits.or.nausea	NO	NO	NO	NO	NO	YES	NO
Diarrhea	NO	NO	NO	NO	NO	YES	NO
SAH..Systemic.Arterial.Hypertension.	YES	NO	NO	NO	YES	NO	NO
Cardiovascular.disease..not.SAH.	NO	NO	NO	NO	YES	NO	NO
Obesity	YES	YES	NO	NO	NO	NO	NO
DM..Diabetes.Melitus.	NO	NO	NO	NO	YES	NO	NO
Neoplasias	YES	NO	NO	NO	NO	NO	NO
Lung.Disease	NO	NO	NO	YES	YES	NO	NO
COPD..Chronic.Obstrutive.Pulmonar.Disease.	NO	NO	NO	NO	YES	NO	NO
Smoker.or.Ex.Smoker.	NO	NO	NO	NO	NO	NO	NO
Asthma	NO	NO	NO	NO	NO	NO	NO
Kidney.disease	NO	NO	YES	NO	NO	NO	NO
Ground.glass.Opacity	YES	YES	YES	YES	NO	YES	YES
Pulmonary.Commitment.Degree	NO	NO	YES	YES	NO	YES	YES
Suggestive.of.viral.infection	YES	YES	YES	YES	NO	YES	YES

Supplementary Table S6B: Classification of the Withheld Set 2 patients and their clinical characteristics.

**Patient ID #**	**52**	**95**	**10**	**91**	**198**	**269**	**56**
**Clinical Classification**	NEG_H	NEG_H	NEG_H	NEG_H	NEG_H	NEG_H	NEG_H
**Model Prediction Result**	POS_H	POS_H	POS_H	POS_H	POS_H	POS_H	POS_H
Gender	F	M	F	M	F	M	F
Age	69	43	60	64	75	66	45
RT.PCR	NEG	NEG	NEG	NEG	NEG	NEG	NEG
Days.in.between.PCR.and.MS.sample.collection	3	7	5	6	6	6	
MS.Collection.Symptoms.onset.difference	-10	-9	-20	-26	-9	-10	-14
Mechanical.ventilation..days.	0	0	0	1	N/A	0	0
Fever	NO	YES	NO	YES	YES	NO	YES
Cough	YES	NO	YES	NO	NO	NO	YES
Myalgia	NO	NO	NO	NO	NO	NO	YES
Sore.Throat	NO	NO	NO	YES	NO	NO	NO
Headache	NO	NO	YES	NO	NO	NO	NO
Coryza	NO	NO	NO	YES	NO	NO	NO
Dyspnea	YES	NO	YES	NO	NO	YES	NO
SatO2.95	NO	NO	NO	YES	NO	YES	NO
Tiredness.fatigue.	YES	NO	NO	NO	NO	NO	NO
Loss.of.smell.or.taste	NO	NO	NO	YES	YES	NO	NO
Vomits.or.nausea	NO	NO	NO	NO	NO	NO	NO
Diarrhea	NO	NO	NO	YES	NO	NO	NO
SAH..Systemic.Arterial.Hypertension.	YES	NO	NO	YES	YES	NO	NO
Cardiovascular.disease..not.SAH.	YES	NO	YES	NO	NO	NO	NO
Obesity	NO	NO	YES	NO	NO	NO	NO
DM..Diabetes.Melitus.	YES	NO	NO	NO	YES	NO	NO
Neoplasias	NO	NO	NO	NO	NO	NO	NO
Lung.Disease	YES	NO	NO	NO	NO	NO	NO
COPD..Chronic.Obstrutive.Pulmonar.Disease.	YES	NO	NO	NO	NO	NO	NO
Smoker.or.Ex.Smoker.	YES	NO	NO	YES	NO	YES	NO
Asthma	NO	NO	NO	NO	NO	NO	NO
Kidney.disease	NO	NO	NO	NO	NO	NO	NO
Ground.glass.Opacity	YES	YES	YES	YES	YES	NO	NO
Pulmonary.Commitment.Degree	NO	YES	YES	YES	YES	NO	NO
Suggestive.of.viral.infection	YES	YES	YES	YES	YES	NO	NO

Supplementary Table S6B: Classification of the Withheld Set 2 patients and their clinical characteristics.

**Patient ID #**	**22**	**115**	**159**	**82**	**150**	**61**	**157**
**Clinical Classification**	NEG_H	NEG_H	NEG_H	NEG_H	NEG_H	POS_H	POS_H
**Model Prediction Result**	POS_H	POS_H	POS_H	POS_H	POS_H	POS_H	POS_H
Gender	F	F	M	M	M	M	F
Age	50	66	30	67	52	55	46
RT.PCR	NEG	NEG	NEG	NEG	NEG	POS	POS
Days.in.between.PCR.and.MS.sample.collection	8	7	-4	3	3	4	11
MS.Collection.Symptoms.onset.difference	-10	-9	-15	-8	-10	-7	-18
Mechanical.ventilation..days.	0	0	0	0	0	N/A	0
Fever	NO	NO	YES	YES	YES	NO	YES
Cough	YES	YES	NO	NO	NO	YES	NO
Myalgia	YES	NO	NO	NO	YES	NO	NO
Sore.Throat	NO	NO	NO	NO	YES	NO	NO
Headache	NO	NO	YES	YES	NO	NO	NO
Coryza	NO	NO	NO	NO	YES	NO	NO
Dyspnea	YES	YES	YES	NO	NO	YES	NO
SatO2.95	YES	NO	NO	NO	NO	NO	NO
Tiredness.fatigue.	NO	NO	NO	NO	YES	NO	NO
Loss.of.smell.or.taste	NO	NO	NO	NO	YES	NO	NO
Vomits.or.nausea	NO	NO	NO	NO	NO	NO	NO
Diarrhea	NO	NO	NO	NO	YES	NO	YES
SAH..Systemic.Arterial.Hypertension.	NO	YES	NO	YES	YES	YES	YES
Cardiovascular.disease..not.SAH.	NO	YES	NO	NO	NO	NO	NO
Obesity	NO	NO	NO	NO	YES	NO	NO
DM..Diabetes.Melitus.	NO	YES	NO	NO	NO	YES	NO
Neoplasias	NO	NO	NO	NO	NO	NO	NO
Lung.Disease	NO	NO	YES	NO	NO	NO	NO
COPD..Chronic.Obstrutive.Pulmonar.Disease.	NO	NO	NO	NO	NO	NO	NO
Smoker.or.Ex.Smoker.	NO	NO	NO	NO	NO	NO	NO
Asthma	NO	NO	YES	NO	NO	NO	NO
Kidney.disease	NO	NO	NO	NO	NO	NO	YES
Ground.glass.Opacity	YES	YES	YES	YES	YES	YES	YES
Pulmonary.Commitment.Degree	YES	YES	NO	YES	YES	YES	YES
Suggestive.of.viral.infection	YES	YES	YES	YES	YES	YES	YES

Supplementary Table S6B: Classification of the Withheld Set 2 patients and their clinical characteristics.

**Patient ID #**	**94**	**1**	**42**	**59**	**106**	**109**	**107**
**Clinical Classification**	POS_H	POS_H	POS_H	POS_H	POS_H	POS_H	POS_H
**Model Prediction Result**	POS_H	POS_H	POS_H	POS_H	POS_H	POS_H	POS_H
Gender	M	M	M	M	M	F	F
Age	28	56	75	77	62	72	62
RT.PCR	POS	POS	POS	POS	POS	POS	POS
Days.in.between.PCR.and.MS.sample.collection	6	8	1	4	15	9	6
MS.Collection.Symptoms.onset.difference	-11	-22	-16	-10	-14	-13	-16
Mechanical.ventilation..days.	0	0	0	18	0	0	0
Fever	YES	YES	YES	YES	NO	YES	NO
Cough	NO	YES	NO	YES	NO	NO	YES
Myalgia	NO	NO	NO	YES	NO	NO	NO
Sore.Throat	NO	NO	YES	YES	NO	NO	NO
Headache	NO	NO	NO	YES	NO	NO	NO
Coryza	NO	NO	NO	NO	NO	NO	NO
Dyspnea	NO	YES	YES	NO	YES	NO	YES
SatO2.95	YES	YES	NO	YES	YES	NO	YES
Tiredness.fatigue.	NO	NO	NO	NO	NO	NO	NO
Loss.of.smell.or.taste	YES	NO	NO	NO	NO	NO	NO
Vomits.or.nausea	NO	YES	NO	NO	NO	NO	NO
Diarrhea	NO	NO	NO	NO	NO	NO	NO
SAH..Systemic.Arterial.Hypertension.	NO	YES	NO	NO	NO	YES	YES
Cardiovascular.disease..not.SAH.	NO	YES	NO	NO	YES	YES	NO
Obesity	NO	NO	NO	NO	NO	YES	NO
DM..Diabetes.Melitus.	NO	NO	NO	NO	NO	NO	NO
Neoplasias	NO	NO	NO	YES	NO	NO	NO
Lung.Disease	NO	NO	YES	NO	NO	YES	NO
COPD..Chronic.Obstrutive.Pulmonar.Disease.	NO	NO	YES	NO	NO	NO	NO
Smoker.or.Ex.Smoker.	NO	NO	NO	NO	NO	NO	NO
Asthma	NO	NO	NO	NO	NO	NO	NO
Kidney.disease	NO	NO	NO	NO	NO	NO	NO
Ground.glass.Opacity	YES	YES	YES	YES	YES	YES	YES
Pulmonary.Commitment.Degree	YES	YES	NO	YES	YES	YES	YES
Suggestive.of.viral.infection	YES	YES	YES	YES	YES	YES	YES

Supplementary Table S6B: Classification of the Withheld Set 2 patients and their clinical characteristics.

**Patient ID #**	**17**	**24**	**133**	**196**	**6**	**86**	**76**
**Clinical Classification**	POS_H	POS_H	POS_H	POS_H	POS_H	POS_H	POS_H
**Model Prediction Result**	POS_H	POS_H	POS_H	NEG_NH	POS_H	POS_H	POS_H
Gender	M	M	M	F	M	M	F
Age	74	68	44	52	55	50	26
RT.PCR	POS	POS	POS	POS	POS	POS	POS
Days.in.between.PCR.and.MS.sample.collection	1	10	4	16	10	13	4
MS.Collection.Symptoms.onset.difference	N/A	-16	-6	-16	-19	-23	-10
Mechanical.ventilation..days.	0	0	0	0	0	0	0
Fever	NO	NO	NO	NO	NO	NO	YES
Cough	YES	YES	YES	YES	YES	YES	NO
Myalgia	YES	NO	NO	NO	NO	NO	NO
Sore.Throat	NO	NO	NO	NO	NO	NO	NO
Headache	NO	NO	NO	YES	NO	NO	NO
Coryza	NO	NO	NO	NO	NO	NO	YES
Dyspnea	NO	YES	YES	NO	YES	YES	NO
SatO2.95	NO	NO	YES	NO	NO	NO	NO
Tiredness.fatigue.	NO	NO	YES	NO	NO	NO	NO
Loss.of.smell.or.taste	NO	NO	YES	YES	NO	NO	NO
Vomits.or.nausea	NO	YES	NO	NO	NO	NO	NO
Diarrhea	NO	NO	NO	NO	NO	NO	NO
SAH..Systemic.Arterial.Hypertension.	YES	YES	YES	YES	NO	NO	YES
Cardiovascular.disease..not.SAH.	NO	NO	NO	NO	NO	NO	YES
Obesity	NO	YES	YES	YES	NO	NO	NO
DM..Diabetes.Melitus.	YES	NO	YES	NO	YES	NO	NO
Neoplasias	NO	NO	NO	NO	NO	NO	NO
Lung.Disease	NO	NO	NO	NO	NO	NO	NO
COPD..Chronic.Obstrutive.Pulmonar.Disease.	NO	NO	NO	NO	NO	NO	NO
Smoker.or.Ex.Smoker.	NO	NO	NO	NO	YES	NO	NO
Asthma	NO	NO	NO	NO	NO	YES	NO
Kidney.disease	NO	NO	NO	NO	NO	NO	NO
Ground.glass.Opacity	YES	YES	YES	YES	YES	YES	YES
Pulmonary.Commitment.Degree	YES	YES	YES	YES	YES	YES	YES
Suggestive.of.viral.infection	YES	YES	YES	YES	YES	YES	YES

Supplementary Table S6B: Classification of the Withheld Set 2 patients and their clinical characteristics.

**Patient ID #**	**143**	**103**	**83**	**16**	**5**	**50**	**81**
**Clinical Classification**	POS_H	POS_H	POS_H	POS_H	POS_H	POS_H	POS_H
**Model Prediction Result**	POS_H	POS_H	POS_H	POS_H	POS_H	POS_H	POS_H
Gender	M	F	M	M	M	F	M
Age	37	62	62	35	60	65	77
RT.PCR	POS	POS	POS	POS	POS	POS	POS
Days.in.between.PCR.and.MS.sample.collection	5	8	3	3	6	3	8
MS.Collection.Symptoms.onset.difference	-10	-12	-3	-13	-13	-11	-14
Mechanical.ventilation..days.	0	0	0	0	0	0	0
Fever	YES	YES	YES	YES	NO	YES	YES
Cough	NO	NO	NO	YES	NO	YES	NO
Myalgia	NO	NO	NO	NO	YES	YES	NO
Sore.Throat	NO	NO	NO	NO	NO	NO	NO
Headache	NO	NO	NO	NO	NO	NO	NO
Coryza	NO	NO	NO	NO	NO	NO	NO
Dyspnea	NO	NO	NO	YES	YES	NO	NO
SatO2.95	NO	NO	YES	NO	NO	YES	NO
Tiredness.fatigue.	NO	NO	NO	NO	NO	YES	NO
Loss.of.smell.or.taste	NO	NO	NO	NO	YES	NO	NO
Vomits.or.nausea	NO	YES	YES	NO	NO	NO	NO
Diarrhea	NO	YES	NO	NO	NO	NO	NO
SAH..Systemic.Arterial.Hypertension.	NO	NO	YES	NO	YES	YES	YES
Cardiovascular.disease..not.SAH.	NO	NO	NO	NO	NO	NO	NO
Obesity	NO	NO	YES	NO	NO	YES	YES
DM..Diabetes.Melitus.	NO	NO	NO	NO	NO	NO	YES
Neoplasias	NO	NO	NO	NO	NO	NO	NO
Lung.Disease	NO	NO	NO	NO	NO	NO	NO
COPD..Chronic.Obstrutive.Pulmonar.Disease.	NO	NO	NO	NO	NO	NO	NO
Smoker.or.Ex.Smoker.	NO	NO	NO	NO	NO	NO	NO
Asthma	NO	NO	NO	NO	NO	NO	NO
Kidney.disease	NO	NO	NO	NO	NO	NO	NO
Ground.glass.Opacity	YES	YES	YES	YES	YES	YES	YES
Pulmonary.Commitment.Degree	YES	YES	YES	YES	YES	YES	YES
Suggestive.of.viral.infection	YES	YES	YES	YES	YES	YES	YES

Supplementary Table S6B: Classification of the Withheld Set 2 patients and their clinical characteristics.

**Patient ID #**	**89**	**25**	**154**	**85**	**98**	**14**	**160**
**Clinical Classification**	POS_H	POS_H	POS_H	POS_H	POS_H	POS_H	POS_H
**Model Prediction Result**	POS_H	POS_H	POS_H	POS_H	POS_H	POS_H	NEG_NH
Gender	M	M	F	F	M	F	F
Age	47	68	58	46	66	59	46
RT.PCR	POS	POS	POS	POS	POS	POS	POS
Days.in.between.PCR.and.MS.sample.collection	21	10	3	0	10	3	14
MS.Collection.Symptoms.onset.difference	-25	-19	-10	-15	-9	-10	-17
Mechanical.ventilation..days.	4	0	0	0	0	0	0
Fever	YES	NO	YES	YES	NO	YES	YES
Cough	NO	NO	NO	NO	YES	NO	NO
Myalgia	NO	NO	NO	NO	NO	YES	NO
Sore.Throat	NO	NO	NO	NO	NO	NO	NO
Headache	NO	NO	YES	NO	NO	NO	NO
Coryza	NO	NO	YES	YES	NO	NO	NO
Dyspnea	NO	YES	NO	NO	YES	YES	NO
SatO2.95	YES	YES	NO	NO	NO	NO	NO
Tiredness.fatigue.	YES	NO	YES	NO	NO	NO	NO
Loss.of.smell.or.taste	NO	NO	NO	NO	NO	YES	NO
Vomits.or.nausea	NO	NO	NO	NO	NO	NO	NO
Diarrhea	NO	NO	YES	NO	NO	NO	NO
SAH..Systemic.Arterial.Hypertension.	NO	YES	NO	NO	YES	NO	NO
Cardiovascular.disease..not.SAH.	NO	NO	NO	NO	NO	NO	NO
Obesity	NO	NO	NO	NO	NO	YES	NO
DM..Diabetes.Melitus.	NO	NO	NO	NO	YES	YES	NO
Neoplasias	NO	NO	NO	NO	NO	NO	NO
Lung.Disease	NO	NO	NO	NO	NO	NO	NO
COPD..Chronic.Obstrutive.Pulmonar.Disease.	NO	NO	NO	NO	NO	NO	NO
Smoker.or.Ex.Smoker.	NO	NO	NO	NO	NO	NO	NO
Asthma	NO	NO	NO	NO	NO	NO	NO
Kidney.disease	NO	NO	NO	NO	NO	NO	NO
Ground.glass.Opacity	YES	YES	YES	YES	YES	YES	YES
Pulmonary.Commitment.Degree	YES	YES	YES	YES	YES	NO	YES
Suggestive.of.viral.infection	YES	YES	YES	YES	YES	YES	YES

Supplementary Table S6B: Classification of the Withheld Set 2 patients and their clinical characteristics.

**Patient ID #**	**104**	**190**	**237**	**90**	**75**	**51**	**4**
**Clinical Classification**	POS_H	POS_H	POS_H	POS_H	POS_H	POS_H	POS_H
**Model Prediction Result**	NEG_NH	POS_H	POS_H	POS_H	POS_H	NEG_NH	POS_H
Gender	F	F	M	F	F	F	M
Age	51	46	76	68	69	72	47
RT.PCR	POS	POS	POS	POS	POS	POS	POS
Days.in.between.PCR.and.MS.sample.collection	7	3	5	18	4	21	2
MS.Collection.Symptoms.onset.difference	-16	-13	-15	-25	-8	-10	-16
Mechanical.ventilation..days.	0	0	0	0	0	0	0
Fever	YES	YES	NO	YES	NO	NO	YES
Cough	NO	YES	YES	NO	YES	YES	YES
Myalgia	NO	NO	NO	NO	YES	NO	YES
Sore.Throat	NO	YES	NO	NO	NO	NO	NO
Headache	NO	NO	NO	YES	NO	NO	YES
Coryza	NO	NO	NO	YES	NO	NO	YES
Dyspnea	NO	YES	YES	NO	NO	YES	NO
SatO2.95	NO	NO	YES	NO	NO	YES	YES
Tiredness.fatigue.	NO	YES	NO	NO	NO	NO	NO
Loss.of.smell.or.taste	NO	YES	YES	NO	YES	NO	NO
Vomits.or.nausea	YES	NO	NO	YES	NO	NO	NO
Diarrhea	NO	NO	NO	YES	NO	NO	YES
SAH..Systemic.Arterial.Hypertension.	YES	NO	YES	YES	YES	YES	NO
Cardiovascular.disease..not.SAH.	NO	NO	YES	YES	NO	NO	YES
Obesity	YES	NO	NO	NO	YES	NO	NO
DM..Diabetes.Melitus.	NO	NO	YES	YES	YES	YES	YES
Neoplasias	NO	NO	NO	NO	NO	NO	NO
Lung.Disease	YES	NO	NO	NO	NO	YES	NO
COPD..Chronic.Obstrutive.Pulmonar.Disease.	NO	NO	NO	NO	NO	YES	NO
Smoker.or.Ex.Smoker.	NO	NO	NO	NO	NO	NO	NO
Asthma	YES	NO	NO	NO	NO	NO	NO
Kidney.disease	NO	NO	NO	NO	NO	NO	NO
Ground.glass.Opacity	YES	YES	YES	YES	YES	YES	YES
Pulmonary.Commitment.Degree	YES	YES	YES	YES	YES	NO	YES
Suggestive.of.viral.infection	YES	YES	YES	YES	YES	YES	YES

Supplementary Table S6B: Classification of the Withheld Set 2 patients and their clinical characteristics.

**Patient ID #**	**67**	**3**	**57**	**15**	**139**	**99**	**19**
**Clinical Classification**	POS_H	POS_H	POS_H	POS_H	POS_H	POS_H	POS_H
**Model Prediction Result**	POS_H	POS_H	POS_H	POS_H	NEG_NH	POS_H	POS_H
Gender	M	M	M	F	M	F	M
Age	65	55	40	59	49	53	54
RT.PCR	POS	POS	POS	POS	POS	POS	POS
Days.in.between.PCR.and.MS.sample.collection	3	4	8	13	3	11	-
MS.Collection.Symptoms.onset.difference	-10	-7	-13	-26	-8	-11	-13
Mechanical.ventilation..days.	0	0	0	0	0	0	0
Fever	YES	NO	YES	NO	NO	NO	YES
Cough	YES	YES	YES	YES	YES	YES	NO
Myalgia	YES	NO	NO	NO	YES	NO	NO
Sore.Throat	NO	NO	NO	NO	YES	NO	NO
Headache	NO	NO	YES	NO	NO	NO	NO
Coryza	NO	NO	NO	NO	YES	NO	NO
Dyspnea	NO	YES	YES	YES	NO	YES	YES
SatO2.95	NO	NO	NO	YES	NO	NO	NO
Tiredness.fatigue.	NO	NO	NO	NO	NO	NO	NO
Loss.of.smell.or.taste	YES	NO	NO	YES	NO	NO	NO
Vomits.or.nausea	NO	NO	NO	NO	NO	NO	NO
Diarrhea	YES	YES	NO	YES	NO	NO	NO
SAH..Systemic.Arterial.Hypertension.	YES	NO	NO	YES	NO	NO	YES
Cardiovascular.disease..not.SAH.	YES	NO	NO	NO	NO	NO	NO
Obesity	NO	NO	NO	NO	NO	NO	YES
DM..Diabetes.Melitus.	NO	NO	NO	NO	NO	NO	YES
Neoplasias	NO	NO	NO	NO	NO	NO	NO
Lung.Disease	NO	NO	NO	NO	NO	NO	NO
COPD..Chronic.Obstrutive.Pulmonar.Disease.	NO	NO	NO	NO	NO	NO	NO
Smoker.or.Ex.Smoker.	YES	NO	NO	NO	YES	NO	NO
Asthma	NO	NO	NO	NO	NO	NO	NO
Kidney.disease	NO	NO	NO	NO	NO	NO	NO
Ground.glass.Opacity	NO	YES	YES	YES	NO	YES	YES
Pulmonary.Commitment.Degree	NO	YES	YES	YES	NO	YES	NO
Suggestive.of.viral.infection	NO	YES	YES	YES	NO	YES	YES

Supplementary Table S6B: Classification of the Withheld Set 2 patients and their clinical characteristics.

**Patient ID #**	**11**	**197**	**64**	**47**	**134**	**23**	**18**
**Clinical Classification**	POS_H	POS_H	POS_H	POS_H	POS_H	POS_H	POS_H
**Model Prediction Result**	POS_H	POS_H	POS_H	POS_H	POS_H	NEG_NH	POS_H
Gender	F	M	F	M	M	F	M
Age	29	59	58	47	53	53	39
RT.PCR	POS	POS	POS	POS	POS	POS	POS
Days.in.between.PCR.and.MS.sample.collection	4	20	2	-	8	24	-
MS.Collection.Symptoms.onset.difference	-7	-29	-16	-11	-28	-28	-11
Mechanical.ventilation..days.	0	0	0	0	0	6	0
Fever	YES	YES	YES	YES	YES	YES	YES
Cough	YES	NO	YES	YES	NO	YES	YES
Myalgia	NO	NO	YES	NO	NO	NO	YES
Sore.Throat	YES	NO	YES	NO	NO	NO	NO
Headache	NO	NO	NO	NO	NO	NO	NO
Coryza	NO	NO	NO	NO	NO	NO	NO
Dyspnea	YES	NO	NO	YES	NO	YES	NO
SatO2.95	YES	YES	YES	NO	NO	YES	NO
Tiredness.fatigue.	NO	YES	NO	NO	YES	NO	NO
Loss.of.smell.or.taste	NO	YES	NO	NO	NO	YES	NO
Vomits.or.nausea	NO	NO	YES	YES	NO	NO	NO
Diarrhea	NO	NO	YES	YES	NO	NO	NO
SAH..Systemic.Arterial.Hypertension.	NO	YES	YES	NO	NO	YES	NO
Cardiovascular.disease..not.SAH.	NO	YES	YES	NO	NO	NO	NO
Obesity	NO	NO	NO	NO	NO	NO	YES
DM..Diabetes.Melitus.	NO	YES	YES	NO	NO	YES	NO
Neoplasias	NO	NO	NO	NO	NO	NO	NO
Lung.Disease	NO	NO	NO	NO	NO	NO	NO
COPD..Chronic.Obstrutive.Pulmonar.Disease.	NO	NO	NO	NO	NO	NO	NO
Smoker.or.Ex.Smoker.	NO	NO	NO	NO	NO	NO	NO
Asthma	NO	NO	NO	NO	NO	NO	NO
Kidney.disease	NO	NO	NO	NO	NO	NO	NO
Ground.glass.Opacity	YES	YES	YES	YES	YES	YES	YES
Pulmonary.Commitment.Degree	YES	NO	YES	YES	YES	YES	YES
Suggestive.of.viral.infection	YES	YES	YES	YES	YES	YES	YES

Supplementary Table S6B: Classification of the Withheld Set 2 patients and their clinical characteristics.

**Patient ID #**	**49**	**63**	**26**	**38**	**62**	**113**
**Clinical Classification**	POS_H	POS_H	POS_H	POS_H	POS_H	POS_H
**Model Prediction Result**	POS_H	POS_H	POS_H	POS_H	POS_H	NEG_NH
Gender	M	F	M	M	F	M
Age	62	71	69	56	61	60
RT.PCR	POS	POS	POS	POS	POS	POS
Days.in.between.PCR.and.MS.sample.collection	4	8	1	7	3	17
MS.Collection.Symptoms.onset.difference	-4	-11	-15	-7	-9	-20
Mechanical.ventilation..days.	0	0	0	0	3	0
Fever	YES	NO	YES	NO	YES	YES
Cough	YES	YES	YES	YES	YES	YES
Myalgia	YES	NO	YES	NO	NO	NO
Sore.Throat	NO	NO	NO	YES	YES	NO
Headache	NO	YES	NO	NO	NO	NO
Coryza	NO	NO	NO	NO	NO	NO
Dyspnea	NO	YES	NO	YES	YES	YES
SatO2.95	NO	NO	NO	NO	NO	YES
Tiredness.fatigue.	NO	NO	NO	NO	NO	NO
Loss.of.smell.or.taste	NO	NO	NO	NO	NO	NO
Vomits.or.nausea	NO	NO	NO	NO	YES	NO
Diarrhea	NO	YES	NO	NO	YES	NO
SAH..Systemic.Arterial.Hypertension.	NO	NO	NO	YES	NO	YES
Cardiovascular.disease..not.SAH.	NO	NO	NO	NO	NO	NO
Obesity	NO	NO	YES	NO	NO	NO
DM..Diabetes.Melitus.	YES	NO	NO	YES	NO	NO
Neoplasias	NO	NO	NO	NO	NO	NO
Lung.Disease	NO	NO	NO	NO	YES	NO
COPD..Chronic.Obstrutive.Pulmonar.Disease.	NO	NO	NO	NO	YES	NO
Smoker.or.Ex.Smoker.	NO	NO	NO	NO	NO	NO
Asthma	NO	NO	NO	NO	NO	NO
Kidney.disease	NO	NO	NO	NO	NO	NO
Ground.glass.Opacity	YES	YES	YES	YES	NO	YES
Pulmonary.Commitment.Degree	YES	YES	YES	YES	NO	YES
Suggestive.of.viral.infection	YES	YES	YES	YES	NO	YES
